# Nanotechnology–General Aspects: A Chemical Reduction Approach to the Synthesis of Nanoparticles

**DOI:** 10.3390/molecules28134932

**Published:** 2023-06-22

**Authors:** Paulina Szczyglewska, Agnieszka Feliczak-Guzik, Izabela Nowak

**Affiliations:** Faculty of Chemistry, Adam Mickiewicz University in Poznań, Uniwersytetu Poznańskiego 8, 61-614 Poznań, Poland; nowakiza@amu.edu.pl

**Keywords:** nanotechnology, metal nanoparticles, chemical reduction

## Abstract

The role of nanotechnology is increasingly important in our society. Through it, scientists are acquiring the ability to understand the structure and properties of materials and manipulate them at the scale of atoms and molecules. Nanomaterials are at the forefront of the rapidly growing field of nanotechnology. The synthesis of nanostructured materials, especially metallic nanoparticles, has attracted tremendous interest over the past decade due to their unique properties, making these materials excellent and indispensable in many areas of human activity. These special properties can be attributed to the small size and large specific surface area of nanoparticles, which are very different from those of bulk materials. Nanoparticles of different sizes and shapes are needed for many applications, so a variety of protocols are required to produce monodisperse nanoparticles with controlled morphology. The purpose of this review is firstly to introduce the reader to the basic aspects related to the field of nanotechnology and, secondly, to discuss metallic nanoparticles in greater detail. This article explains the basic concepts of nanotechnology, introduces methods for synthesizing nanoparticles, and describes their types, properties, and possible applications. Of many methods proposed for the synthesis of metal nanoparticles, a chemical reduction is usually preferred because it is easy to perform, cost-effective, efficient, and also allows control of the structural parameters through optimization of the synthesis conditions. Therefore, a chemical reduction method is discussed in more detail—each factor needed for the synthesis of nanoparticles by chemical reduction is described in detail, i.e., metal precursors, solvents, reducing agents, and stabilizers. The methods that are used to characterize nanomaterials are described. Finally, based on the available literature collection, it is shown how changing the synthesis parameters/methods affects the final characteristics of nanoparticles.

## 1. Basic Aspects of Nanotechnology

### 1.1. The Era of Nanomaterials

As known from history, different periods in the history of civilization have their specific names; for instance, the Stone Age refers to the period from the appearance of the first stone tools used by man, the Bronze Age refers to when tools made from copper-tin alloy were used, and the Iron Age was when iron became the main raw material for making tools [1]. Human dreams combined with imagination often give birth to new sciences—which is how nanotechnology was born. Since nanotechnology is unquestionably one of the top buzzwords of the new millennium and has so far significantly raised the standard of living, this period might be called the era of nanomaterials. Some researchers even go so far as to say that the impact of nanotechnology will be so great that the term will be used to describe a new era of global economic growth [2]. It is predicted that the level of nanotechnology will determine a country’s position in the global economy. Thus, the products of nanotechnology are expected to make a significant contribution to the problem-solving process through the use of smaller materials and systems [3].

**Figure 1:**
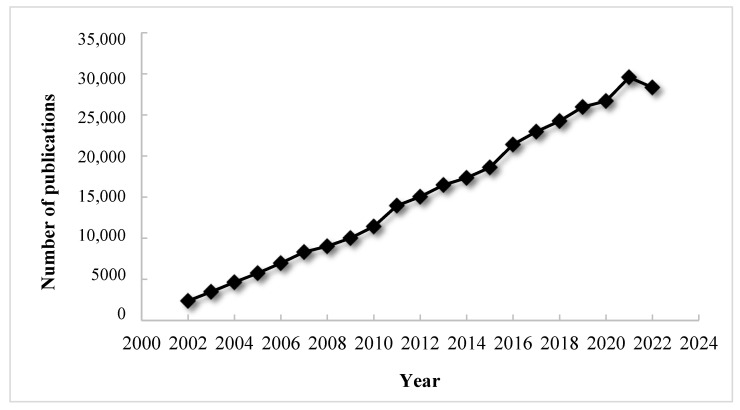
Number of publications in the field of nanotechnology according to the Web of Science (literature items that include the prefix “nano” in the topic); data as of 20 May 2023.

The topics related to nanotechnology have recently attracted a great deal of attention from scientists around the world. Over the past 20 years, the number of literature items related to this subject has experienced tremendous growth, as shown in the chart below (Figure 1). The number of scientific papers with the keyword “nano” is increasing over time in an approximately linear fashion, and it is predicted that in 2025 there will be more than 30,000 of them. This article provides an overview of the basic aspects of nanotechnology, introduces the broad topic of transition metal nanoparticles, discussing their properties and methods of synthesis and characterization. The next section develops the topic of the synthesis of transition metal nanoparticles by chemical reduction and provides an extensive review of relevant literature. The last section also includes a discussion of the effects of various factors on the final nanoparticles produced by the method of chemical reduction.

### 1.2. What Is Nanotechnology?

The answer to this question is not straightforward. It is best to start the explanation with the prefix “nano”, which comes from the Greek word “nanos”, which translates into “dwarf”. Therefore, nanotechnology can simply mean technology related to small things. However, the prefix “nano” also has another meaning—in scientific language, it means 1 billionth of a meter, referred to as a “nanometer” [2,3,4,5]. Given this, nanotechnology can be viewed as being related to technologies operating at the nanometer level. In 1994, the Royal Society/Royal Academy of Engineering Working Group adopted the following definitions to help distinguish between nanoscience and nanotechnology, namely [3,6]:

- nanoscience—is the study of structures and molecules at the atomic, molecular, and macromolecular scales, whose properties are significantly different from those occurring on a larger scale,
- nanotechnology—is technology that uses nanoscience in practical applications, such as a variety of devices or systems, by controlling shape and size at the nanometer scale.

Nanotechnology is thus an interdisciplinary field that integrates science and technology [1,7]. However, it is a very broad discipline, and putting it into a single definition can be misleading. Moreover, there is no limit to the areas of research or end products that will fall under the definition of nanotechnology. So it is worth looking at the basic concepts in the field of nanotechnology as cited below [8]:

- nanoscale—a scale having one or more dimensions of the order of 100 nm or less,
- nanomaterial—a material with one or more external dimensions or internal structure that can exhibit novel properties compared to the same material without nanoscale features,
- nanoparticle—a particle with at least one dimension at the nanoscale,
- nanocomposite—a composite in which at least one of the phases has at least one dimension at the nanoscale,
- nanostructured materials—having a structure at the nanoscale.

The National Nanotechnology Initiative has clarified the definition of nanotechnology and defined nanotechnology as a field that includes [7,9]:

- research and development of technologies in the 1–100 nm range,
- creation of small structures with novel properties,
- controlling and manipulating structures at the atomic scale.

Nanotechnology, therefore, refers to materials and systems whose structures and components exhibit new and significantly changed physical, chemical, and biological properties due to their size at the nanoscale. Materials made of structures of sizes in the range of about 10^−9^–10^−7^ m (1–100 nm) show important changes in the characteristics compared to those of isolated molecules (1 nm) or bulk materials [10,11]. Although nanotechnology is a new word, it is not an entirely new field. Nature has created many objects and processes that operate on the micro to nano scale. The new behavior of nanoparticles at the nanoscale is not necessarily predictable from that observed at large scales. Important changes in behavior are not only caused by order of magnitude reduction but also by new phenomena such as size limitation, the prevalence of interfacial phenomena, quantum mechanics, and Coulomb blocking [10]. It is worth going back a few decades to realize how this amazing field of science, now called nanotechnology, has developed.

### 1.3. Short History of Nanotechnology

The development of nanoscience can be traced as far back as the ancient Greeks in the fifth century BC, when they pondered whether the matter was continuous and, therefore, divided into smaller parts or whether it consisted of small, indivisible particles, which are referred to as atoms [3]. Nevertheless, the term “nanotechnology” was first used by Japanese scientist Norio Taniguchi, who delivered a lecture in 1974 describing how the dimensional accuracy with which things are created has improved over time (1940s–1970s). The scientist correctly predicted that by the late 1980s, techniques would evolve to the point where dimensional accuracies greater than 100 nm would become possible. He advocated that nanotechnology involved the processing, separation, consolidation, and deformation of materials by a single atom or molecule [3,12].

On the other hand, even earlier, in 1959, physicist Richard Feynman (winner of the 1965 Nobel Prize in Physics) gave his famous lecture on “There is plenty of room at the bottom” at a meeting of the American Physical Society, outlining the prospects for atomic engineering concerning structures at a level of single atoms or molecules. Although what he talked about was not explicitly called “nanotechnology,” the ideas he presented were prophetic indeed, and this area is what we call “nanotechnology” today. Among other things, Professor Feynman predicted the development of techniques that could be used to create large integrated circuits and the revolutionary effects that the use of these circuits would produce. For these reasons, he is considered the father of modern nanotechnology [13].

The golden era of nanotechnology did not begin until the 1980s, when the miniaturization of instruments using microfabrication technology began [10]. The greatest fruit of miniaturization and, at the same time, a very big step in the development of nanotechnology as a scientific field was the construction of two types of microscopes: the Scanning Tunneling Microscope in 1982 (Gerd Binnig and Heinrich Rohrer) [14,15] and the Atomic Force Microscope in 1986 (Gerd Binnig, Calvin F. Quate, Christoph Gerber) [16,17], which enabled researchers to see and manipulate atoms for the first time. To this day, many opportunities to study nanostructures have arisen, thereby generating excitement in the scientific community. Scientists from almost all disciplines are eagerly pursuing the production and measurement of nanostructures to see what new interesting phenomena are happening on this scale [10]. In everyday life, although we do not think about it, we may perceive the huge impact of very small-scale processes on our world.

At the beginning of the 21st century, interest in the new fields of nanoscience and nanotechnology increased. In the United States, for example, Feynman’s attitude and his concept of manipulating matter at the atomic level played an important role in shaping national scientific priorities. President Bill Clinton advocated funding research into this emerging technology in a speech at Caltech in 2000. Three years later, President George W. Bush signed into law the 21st Century Nanotechnology Research and Development Act. The legislation made nanotechnology research a national priority and created the National Technology Initiative [13].

### 1.4. Application of Nanotechnology Products

**Figure 2:**
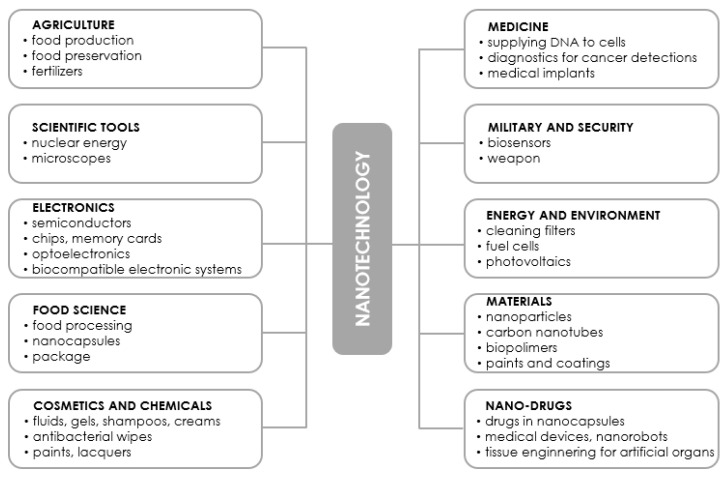
Potential applications of nanotechnology products.

It is not an exaggeration to say that nanotechnology can be a threat to many industries due to the fact that it often offers smaller, cheaper, and faster devices with superior functionality while using less raw materials and energy [1,18]. It has the potential to affect the production of virtually every man-made object, from automobiles, electronics, advanced diagnostics, and surgery, to advanced medicines and tissue/bone substitutes. Today’s nanotechnology takes advantage of current advances in chemistry, physics, materials science, biotechnology, and electronics to create new materials that have unique properties. Some of the applications of nanotechnology have already made their mark on the market, while others are being intensively researched for solutions to humanity’s biggest problems: diseases, clean energy, clean water, etc. The products of advanced nanotechnology that will be available in the coming decades promise even more revolutionary applications than the products of current and near-future nanotechnology [12]. The results of exciting projects are being published at a steady pace in the form of scientific papers, review articles as well as patents, but it is impossible to list or discuss all the areas of activity that would involve the use of nanotechnology products. Nevertheless, the remainder of this literature review describes in very general terms selected areas of application of nanotechnology products. In addition, Figure 2 collects the applications most frequently mentioned in the literature without distinguishing between those already on the market and those in the research phase.

#### 1.4.1. Energy and Environment

The uncertain future of traditional energy sources and increasingly new environmental policies have contributed to a global movement to develop and implement alternative methods of energy extraction and efficient energy use. Nowadays, nanotechnological inventions are penetrating into various subsystems of the entire energy system, starting from energy acquisition, conversion, distribution, and storage and ending with energy utilization [19,20]. The application of nanotechnology in the energy field, which includes lithium-ion batteries, fuel cells, light-emitting diodes, ultracapacitors, and solar cells, among others, is a hot topic in many scientific studies. Unfortunately, the development of this field of nanotechnology has been hampered by significant production costs compared to that of conventional technologies [14]. Today, thanks to nanomaterials, photovoltaic cells are increasing their efficiency while reducing the cost of electricity production at an unprecedented rate. The production, storage, and conversion of hydrogen into electricity in fuel cells benefit from more efficient water-splitting catalysts, better-nanostructured materials for higher hydrogen adsorption capacity, and cheaper yet simpler fuel cells [19].

#### 1.4.2. Electronics

Miniaturization of electronic devices is considered a key factor in achieving higher efficiency and speed of information exchange. This achievement is now available mainly due to great advances in nanolithography techniques. Thanks to the nanotechnology-based approach, the performance of traditional semiconductors has increased, and new possibilities have emerged [19]. Creating more efficient computer processors is another advantage of nanoelectronics, which can improve the capabilities of electronic components in several ways: (i) by improving the screens displayed on electronic devices, which includes reducing power consumption while reducing the weight and thickness of the screens, (ii) by increasing the density of memory chips, and (iii) by reducing the size of transistors used in integrated circuits [21]. Products of nanotechnology are also used in supercapacitors—which are a type of electrochemical capacitors and have been recognized as some of the most reliable and efficient energy storage and conversion devices. For example, nanowires are being produced and used as electrode material for supercapacitors [22,23].

#### 1.4.3. Agri-Food Production

Nanotechnology has been recognized as a valuable tool in modern agriculture, as well as in all areas of the food industry, which include: improved food quality and safety, reduced agricultural inputs, enriched nutrient absorption from the soil, pathogen detection, food processing, and packaging, delivery of bioactive compounds to destinations, and production of nanotechnology-based food additives. New approaches based on nanotechnology lead to increased safety and nutritional value of food products, reduced chemical spreading, minimized nutrient losses in fertilization, and increased yields through pest control [24]. In addition, new agrochemicals and new delivery mechanisms have been developed based on nanotechnological devices to improve crop yields and reduce pesticide use [19,24,25].

#### 1.4.4. Cosmetics

Applications of nanotechnology and nanomaterials can be found in many cosmetic products, including moisturizers, hair care products, makeup products, and sunscreens [26,27]. Novel nanocarriers with various formulations, such as liposomes, niosomes, nanoemulsions, microemulsions, solid lipid nanoparticles, nanostructured lipid carriers, and nanospheres, have replaced the use of conventional delivery systems. These novel nanocarriers have the advantages of increased skin penetration (vitamins and other antioxidants), controlled and prolonged drug release, greater stability (unsaturated fatty acids, vitamins, antioxidants encapsulated in nanoparticles), site targeting, high entrapment efficiency, and improved aesthetics of the final product (e.g., in mineral sunscreens, making the particles of the active mineral smaller; they can be applied without leaving a noticeable white film) [19,25]. However, it should be mentioned that nanotoxicological studies have shown concern about the impact of the increased use of nanoparticles in cosmetics, as there is the potential for them to penetrate the skin and cause health risks [28,29], which is described in more detail later in this article.

#### 1.4.5. Medicine

Since several components of living cells are nanoscale in size, it was foreseeable that nanotechnology would be useful in biology and medicine. Today, some nanoscale materials have found clinical applications, and the field of nanomedicine has emerged as a result. As one of the main parts of nanotechnology, nanomedicine aims to provide more efficient tools for preventing and treating various diseases through the interaction of nanomaterials with biological molecules. Strategies based on nanomedicine have opened up new horizons for biomedical engineers as well as clinicians in the prevention, diagnosis, and treatment of serious diseases. With nanotechnology applied to medicine, significant improvements have been witnessed in drug delivery systems, protein detection, cancer treatment, medical imaging and diagnostic platforms, implantable materials, and tissue regeneration strategies, among others [19,30,31,32]. It should be mentioned that various nanosystems have already been used for the benefit of human health, and a larger number of medical projects are underway.

#### 1.4.6. Military and Security

Looking at nanotechnology in the light of national security, it is the latest means by which military weapons can be reduced in size and weight while maintaining the same performance or even increasing it. The potential use of nanotechnology in military applications encompasses almost every aspect, from civilian applications such as ultralight clothing and footwear for the individual warrior to advanced information-processing electronics that can be embedded in a variety of materials [33]. It is certain that nanotechnology will also be used in the military field to design new weapons with unique properties, such as small self-steering mini-robot missiles and the creation of devices that can collect water in all conditions. With all this, it is important to remember that nanotechnological products have a powerful impact on the world, but if they are used for the wrong purposes, the damage could be irreversible, for dealing with nanotechnology is about testing our humanity, ethics, and knowledge [34].

### 1.5. Risk Associated with Nanotechnology

In addition to the many advantages they offer, it is clear that nanotechnological products are not free of drawbacks. The described field of science involves many, often the same problems as in the introduction of any new technology. Manipulation of matter at the nanoscale often adversely affects the environment and human life [1,8,21]. In their work, many scientists point out the risks, toxicity, and many other hazards that are associated with nanotechnology.

Every person is exposed to nanometer-sized particles; we breathe them in with every breath and ingest them with every drink/meal. In fact, every organism on Earth constantly encounters nanometer-sized entities. The vast majority cause little ill effect and go unnoticed, but sometimes nanometer-sized materials can cause significant harm to the body. The interactions between nanomaterials and cells, animals, humans, and the environment are complex, and much research needs to be done to understand in detail how the physical, chemical, and other properties of nanomaterials affect these interactions, and thus determine the ultimate impact of nanomaterials on health and the environment. Research in recent years has confirmed that nanoscale materials exhibit unexpected toxicity. Nanoparticles are more likely to react with cells and various biological components, such as proteins, and travel through organisms, increasing their chances of interacting to trigger inflammatory and immune responses [35,36].

Thus, it is safe to say that today nanotechnology affects human life every day. The potential benefits are many and varied; however, due to the high human exposure to nanoparticles, there is considerable concern about potential health and environmental risks. This concern has even led to the emergence of additional scientific disciplines, including nanotoxicology and nanomedicine. Nanotoxicology involves the study of the potential negative health effects of nanoparticles. Nanomedicine, which includes sub-disciplines such as tissue engineering, biomaterials, biosensors, and bioimaging, has been developed to study the benefits and risks of nanomaterials used in medicine and medical devices. Some of the potential medical benefits of nanomaterials include improved drug delivery, production of antimicrobial coatings for medical devices, reduced inflammation, improved healing of surgical tissues, and detection of circulating cancer cells. However, due to the lack of reliable toxicity data, the potential impact on human health remains a major concern [13].

As a result, new laws and regulations are emerging all the time regarding the risks of nanoproducts. In Europe, the Registration, Evaluation, and Authorization of Chemicals legislation has been in effect since 2007. New European cosmetics regulations require products containing nanomaterials to be listed on the product labels of finished products. In the United States, on the other hand, the U.S. Food and Drug Administration has issued draft guidelines to help manufacturers determine whether their products use nanomaterials, while the Environmental Protection Agency has issued draft guidelines for nanomaterials use [35].

## 2. Nanoparticles

### 2.1. Unique Features of Nanoparticles

The definition of nanoparticles can vary for different fields and different materials. From the theoretical point of view, they are often called nanoclusters or simply clusters, which are defined as a combination of millions of atoms or molecules, which can be of the same or different types. Nanoparticles can be amorphous or crystalline, and their surfaces can act as carriers. NPs exhibit properties between those of bulk material and atomic or molecular structures. They should be considered a distinct state of matter, such as crystalline forms of nanoparticles (fullerenes and carbon nanotubes) and traditional crystalline solid forms (graphite and diamond) [37]. Nanoparticles are everywhere and of great scientific interest. A bulk material has constant physical properties regardless of its size, but at the nanoscale, this is often not the case. It has been proven many times that several well-characterized bulk materials have the most interesting properties when tested at the nanoscale [38]. Nanotechnology thus produces products with completely new properties, often superior to those of the starting material. Nanoparticles have a high percentage of atoms on the surface, and this is the main feature that differentiates their properties from those of bulk material [35,39]. The most important features of NPs that make them so attractive in materials chemistry and chemical engineering are generally related to the following properties [4,5,40,41,42]:

- a high volume-to-surface ratio, one result of which is the high reactivity of nanometer-sized materials, in which interactions between molecules may easily occur,
- the presence of the surface plasmon resonance effect (this aspect is generally important in optical applications),
- different physical properties with respect to the starting metal; surface energy and melting point are particularly sensitive to nanoparticle size,
- a large number of low-coordination sites on the surface relative to that in the starting material, with remarkable effects on chemical reactivity and catalytic properties,
- easy surface functionalization, which makes them very attractive, especially in nanomedicine for selective drug transport in target organs and tissues.

### 2.2. Types of Nanoparticles

Various classification systems have been created to organize nanometer-sized materials. Nanoparticles can be classified based on their properties, such as shape, size, activity, or the type of materials they are made of, among others [19,32]. The following is a classification of nanoparticles based on the materials from which they are made. Using this criterion, nanoparticles are divided into inorganic (carbon-based, metal and metal oxide, semiconductor, ceramic) and organic (polymeric, derived from biomolecules) [43,44]. Thus, nanomaterials can be classified into the following categories:

#### 2.2.1. Inorganic Nanoparticles

- carbon-based nanoparticles

Nanoparticles of this type are composed entirely of carbon, taking the form of a hollow ellipsoid or tube [10]. This class of materials includes single- and multiwalled carbon nanotubes, graphene, fullerenes, nanofibers, fluorescent carbon quantum dots, and carbon dots. The aforementioned materials are widely used in many scientific fields due to their unique physical, chemical, mechanical, and thermal properties. They are characterized by large surface area, good biocompatibility, low toxicity, and low manufacturing costs, and they may be obtained by using greener synthesis routes [44,45,46].

- metal and metal oxide nanoparticles

Metal nanoparticles, as the name implies, consist solely of metal precursors (the metal atom determines the properties of these nanoparticles), while metal oxide nanoparticles consist of a metal precursor combined with oxygen [37]. The materials in question have a unique and wide range of physicochemical properties. They have improved chemical, electrical, optical, thermal, mechanical, electromagnetic, and surface properties compared to bulk materials. In addition, they offer large surface areas, controlled size and morphology, and simple surface modification [44,45]. For these and many other reasons, they have found applications in such areas as biomedicine, catalysis, and energy harvesting (more on the applications of metal nanoparticles is in the following subsections). Metals commonly used to synthesize such nanoparticles include gold (Au), silver (Ag), platinum (Pt), palladium (Pd), copper (Cu), iron (Fe), lead (Pb), and zinc (Zn) [10,44].

- semiconducting nanoparticles and quantum dots

Semiconducting nanostructures exhibit both metallic and non-metallic properties and are characterized by the occurrence of the quantum confinement (quantum confinement) effect, most often when the particle size is smaller than 10 nm [44,47]. The semiconducting nanostructures in which the aforementioned phenomenon occurs are called quantum wells, where the confinement occurs in one dimension, while the nanostructures that are confined in two dimensions are called quantum wires. In contrast, a third type of semiconductor nanostructures that are constrained in three dimensions are quantum dots [46,48]. Semiconducting nanoparticles have found applications in biological research for labeling DNA, cells, and proteins. They are an alternative to natural fluorophores, and their optical properties are controlled by many factors, such as shape, size, doping, and the surrounding environment [44,45].

- ceramic nanoparticles

Ceramic nanoparticles are inorganic solids synthesized by sintering and subsequent cooling [45,46]. They mainly consist of oxides, carbides, phosphates, and carbonates of metals and metalloids, such as calcium, titanium, silicon, etc. Most ceramic nanoparticles are composed of silica or alumina. The porous nature of nanoparticles contributes to their physical protection against degradation and degranulation. Nanophase ceramics can be divided into nanoparticles, nanoshells, and nanoclay [44]. Their special properties, such as high heat resistance, chemical inertness, remarkable mechanical strength, exceptional pH resistance, high loading capacity, and ease of incorporation into hydrophobic and hydrophilic systems, enable their use in various areas, such as catalysis, photocatalysis, and dye photodegradation. They are particularly widely studied in biomedical applications, especially in drug delivery, thanks to their controlled size, surface functionalization, porosity, and surface area-to-volume ratio. However, their disadvantages include low biodegradability, high density, and potential toxicity [44].

#### 2.2.2. Organic Nanoparticles

- polymeric nanoparticles

Polymeric nanoparticles are solid colloidal particles of sizes in the range of 10 nm–1 μm. They are usually made of biodegradable and biocompatible, naturally occurring polymers [44,47]. As a result, they are very often used as drug carriers on whose surface the former can be adsorbed physically or chemically. They are excellent carriers thanks to their small size, water solubility, non-toxicity, long shelf life, and excellent stability. There are two types of polymeric nanoparticles: nanospheres—nanoparticles in which the drug is uniformly dispersed on the matrix, and nanocapsules—nanoparticles in which the drug is embedded in a cavity and surrounded by a polymeric membrane. Natural polymers, such as proteins or polysaccharides, as well as synthetic polymers, are often used for the synthesis of polymeric nanoparticles. Such nanoparticles are extremely susceptible to surface modification through chemical processes and thus have excellent pharmacokinetic control. The most notable nanoparticles of this type are polymers made of polylactic acid, gelatin, poly(lactic and glycolic acid) copolymer, and chitosan. Moreover, such polymers can also be coated on the surface of other types of nanoparticles [44,45,49].

- biomolecule derived nanoparticles

Biomolecules such as proteins, nucleic acids, lipids, and polysaccharides have unique characteristics and can be used to prepare nanoparticles. The biomolecule-derived nanoparticles are increasingly in demand mainly because they are biocompatible and biodegradable. In addition, they are readily available and non-immunogenic. In addition to their own unique functions, bioparticles can conjugate with other inorganic nanoparticles to form special hybrids.

## 3. Metallic Nanoparticles

### 3.1. General Methods for Preparing Metallic Nanoparticles

**Figure 3:**
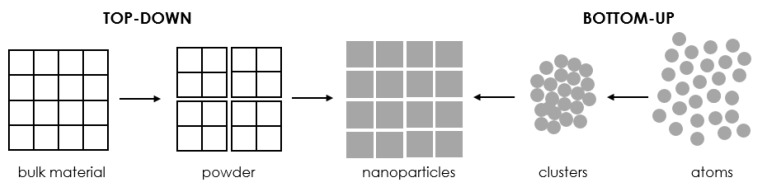
Schematic representation of nanostructure fabrication methods, based on [50].

There are a number of approaches to fabricating nanostructures, which are broadly divided into two main classes, namely bottom-up methods and top-down methods, as schematically presented in Figure 3 [10,11,12,38,40,48,50,51]. These approaches are further subdivided into different subclasses based on operation, response conditions, or other protocols adopted.

#### 3.1.1. The Top-Down Approach

In the top-down approach, the corresponding macroscopic material is reduced by physical or chemical means. In this method, the building blocks atoms or molecules are carefully deposited through controlled reactions, and the self-organization that takes place at a later stage can lead to the formation of nanostructures. The main disadvantage of the top-down approach is the final imperfection of the surface structure. Such defects in surface structure can significantly affect the final physical properties and surface chemistry of metallic nanoparticles due to their high aspect ratio [10,12,32,38,52,53].

#### 3.1.2. The Bottom-Up Approach

The bottom-up approach refers to the construction of an atom-by-atom, molecule-by-molecule, or cluster-by-cluster structure, in which the metal ion is brought to a zero-valent state, and the atoms aggregate further to form nanoparticles [32,40]. It is achieved through processes of self-assembly of individual atoms into larger clusters and their further aggregation into final nanoparticles. In this approach, nanostructured blocks are initially formed and then assembled using chemical or biological procedures. A distinct advantage of the bottom-up approach is the increased possibility of obtaining metallic nanoparticles with a much smaller number of defects and a more homogeneous chemical composition [10,12,38,52,53].

The proposed methods for producing nanostructures are the first rough classification. In order to make the viewer aware of how many methods of nanoparticle synthesis have already been developed, the methods for synthesis of metallic nanoparticles by chemical, physical as well as biological routes have been further divided, which is presented later in this paper.

### 3.2. Methods for Producing Metallic Nanoparticles

#### 3.2.1. Chemical Methods

Chemical methods for the synthesis of metallic nanoparticles are the most widespread, the most numerous, and at the same time, the most efficient ones. These methods are described as easy, convenient, inexpensive (for large-scale production), and quick to carry out while not requiring the use of complex apparatus. Moreover, the final nanoparticles can be stored for long periods of time without significant loss in stability [54]. Although the production of nanoparticles by chemical methods has many advantages, it is pointed out that the use of toxic chemicals necessary for nanoparticle stabilization, as well as solvents, is environmentally unfriendly [47]. In addition, contamination of the final nanoparticles with chemicals is often observed, and significant amounts of hazardous by-products are produced [5,38,55].

#### 3.2.2. Physical Methods

Physical methods for the synthesis of metallic nanoparticles are based on the use of microwaves, ultrasound, irradiation, or mechanical grinding, among others, to obtain the desired product, which involves enormous energy consumption (the need to maintain high pressures and temperatures in most techniques) [5,38,54]. In physical methods, there is no problem of contamination of the final nanoparticles with solvent, which is the case in most chemical methods. Moreover, hazardous materials and chemical reagents are not used in procedures of this type. To sum up, the production rate of nanoparticles is quite low, while the production cost is high [38,54,55].

#### 3.2.3. Biological Methods (Biochemical)

Biological methods are based on the use of a number of biological systems that have the ability to convert metal ions into metal nanoparticles due to the reduced abilities of proteins and metabolites present in these systems [47,54]. Biological methods for the synthesis of nanoparticles involving microorganisms, natural plant extracts, bacterial extracts, enzymes, and plants or plant extracts have been proposed as possible ecological alternatives to chemical and physical methods [56,57,58,59]. Interactions between microorganisms and metals have already been well documented, and the ability of microorganisms to extract/accumulate metals has already been employed in many biotechnological processes, such as bioleaching and bioremediation [60]. Synthesis of nanoparticles by biological methods is easy, cost-effective, energy-efficient (most bioprocesses occur under normal pressure and at ambient temperature), utilizes natural resources, and, with all this, they are environmentally friendly methods that do not require the use of harsh, toxic, and expensive chemicals [38,55,57,61,62].

**Figure 4:**
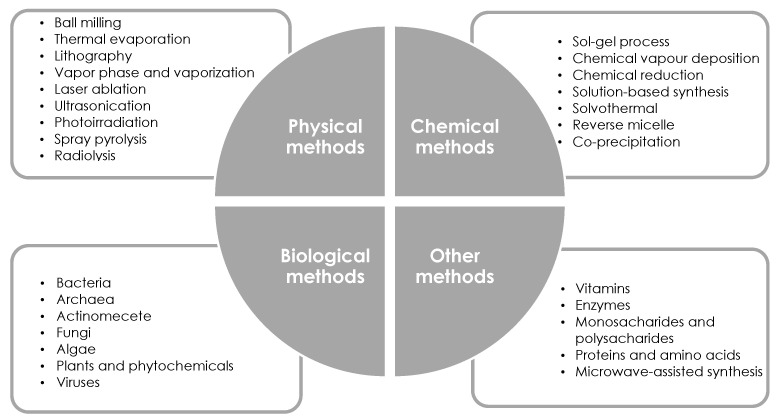
Selected important examples of chemical, physical, biological, and other methods for the preparation of metallic nanoparticles based on [54].

As shown in Figure 4, the above-mentioned types of methods for nanoparticle production, i.e., physical, chemical, and biological, are further divided into many subgroups. In addition, there are still methods that cannot be included in the three described groups—they have been collected in a group labeled as “other methods”. It is impossible to discuss each of the listed methods for synthesizing metallic nanoparticles. The following part of this paper focuses on chemical reduction, which is the most commonly used method for producing metallic nanoparticles.

### 3.3. Synthesis of Metallic Nanoparticles by Chemical Reduction

Metallic nanoparticles have gained a lot of attention because of their unusual properties, which are different from metal in their standard form (bulk metal). The chemical and physical properties of metal nanoparticles strongly depend on their size as well as their structure, shape, and size distribution. Therefore, control of these parameters is crucial and is often achieved by varying synthesis methods, reducing agents, and stabilizers. Precise control of the above parameters allows the achievement of the desired physical and chemical properties changes in nanoparticles.

Chemical reduction, as the name implies, uses chemical-reducing agents. This method can be further classified according to the energy source used or the device used for the reaction, giving a wide range of possibilities for their manufacture [63]. Of the wide range of synthesis methods by chemical means, chemical reduction is the most common and simplest method for synthesizing nanoparticles. The substrates in this method can be either natural compounds from plants or microorganisms or reagents/chemicals that have the ability to cause a reduction in an oxidized state. Moreover, nanoparticles synthesized from natural compounds are less toxic than those prepared using chemicals. An important aspect of the synthesis of nanoparticles by the chemical reduction method is that their size can be strictly controlled, allowing the synthesis of nanoparticles with different morphologies. Further, the method is cost-effective and can be easily scaled up for large-scale preparation without the need for high pressure, energy, and temperature [64].

Because of its simplicity, the main chemical method for the synthesis of metal nanoparticles is the reduction in metal ions in solution (chemical reduction method) [65]. As early as 1857, Michael Faraday was the first to report systematic research on the synthesis of colloidal gold using the chemical reduction method [12]. The chemical reduction process can be carried out in both aqueous and organic solvents, the latter being the preferred choice since metal nanoparticles are particularly sensitive to oxidation [40]. By controlling the reaction parameters, it is possible to produce metal nanoparticles with designer sizes, shapes, and particle size distributions [65]. Parameters affecting the final shape, size, stability, or aggregation state of metallic nanoparticles are listed in Table 1.

**Figure 5:**
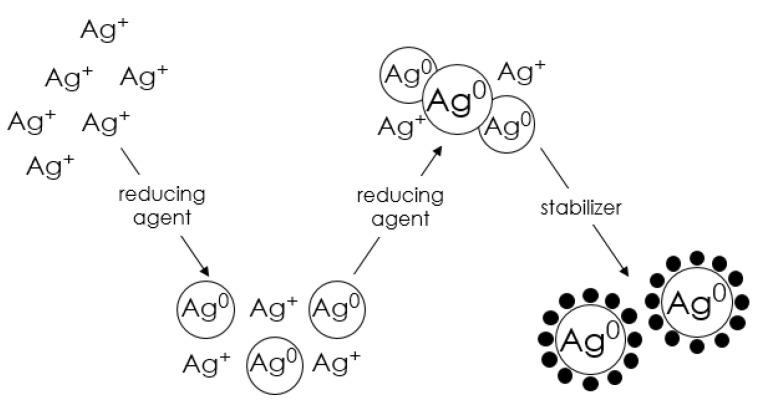
Scheme of nanoparticle formation (using silver as an example) by chemical reduction; based on [60].

The production of metal nanoparticles by chemical reduction involves the reduction of salts of a selected metal by a reducing agent in the presence of a stabilizer [37]. The role of the stabilizer is to protect the metal nanoparticles from assembling into larger aggregates. A typical synthesis of nanoparticles consists of three major steps, as shown in Figure 5. In the first step, a redox reaction takes place, in which electrons from the reducing agent are transferred to the metal atoms, resulting in the formation of free metal atoms. The equation describing the transfer of electrons from the reducing agent to the metal is as follows [66]:

*m*Me^n+^ + *n*Red → *m*Me^0^ + *n*Ox (1)

In the second stage, a nucleation process takes place, in which free metal atoms collide with one another, leading to the formation of stable nuclei. In the last—third stage, stabilizers are added, which prevents the aggregation of nanoparticles.

Metal atoms produced by the reduction in homogeneous solutions are essentially insoluble in the liquid and, therefore, gradually aggregate into clusters called nuclei. The nuclei are dynamic entities involved in a continuous dissociation-condensation process. While new metal atoms are produced in the system, the nuclei reach a critical size and separate from the solution as solid particles (nuclei). The number and size of nuclei produced depend on a number of reaction parameters, such as solute concentration, the redox potential of the reduction reaction, temperature, nature and concentration of the surfactant, solvent viscosity, and surface tension. Nucleation is rarely the final step in the formation of metal particles unless special steps are taken (e.g., by adding special agents). It should be noted that after further addition of metal atoms, nuclei grow to primary particles (nanosize); however, these are generally unstable [66]. In order to produce stable end products, the aggregation process must be stopped at the early stages of particle formation, which can be achieved in a number of ways, namely by electrostatic, steric, electrosteric, and hydration stabilization mechanisms. The following is a description of the necessary components involved in the formation of transition metal nanoparticles, such as metal precursor, solvent, reductant, and stabilizer, giving their role in the process and listing the most commonly used chemical compounds performing the above-described functions.

#### 3.3.1. Metal Precursor

A precursor is a molecule containing the metal atoms from which a nanoparticle will be built and thus is a key factor determining the final nanomaterial. According to a commonly accepted technique, the metal precursor dissolved in a suitable solvent is mixed with both a suitable reducing agent and a suitable stabilizing agent in a well-stirred reactor in an inert atmosphere [40]. The composition of the overall mixture depends on the operating conditions under which the reaction takes place [40]. Based on a review of literature reports in the area of transition metal nanoparticle synthesis, the most common metal precursors used in the synthesis of the most popular metallic nanoparticles, such as Pd, Pt, Ag, Au, Ru, and Cu, are summarized in Table 2.

**Table 2:** Most commonly used precursors in the synthesis of selected transition metal nanoparticles.

Metal	Metal Precursor	References
Pd	Pd(OAc)_2_	[67,68]
	Na_2_PdCl_4_	[69,70,71,72,73]
	Pd(NO_3_)_2_·2H_2_O	[74,75]
	K_2_PdCl_4_	[76]
	H_2_PdCl_4_	[77,78]
	PdCl_2_	[79,80,81,82]
Pt	H_2_PtCl_6_·6H_2_O	[67,73,74,79,83]
	K_2_PtCl_6_	[84,85]
	Na_2_PtCl_4_	[86]
	Pt(acac)_2_	[87]
Ag	AgNO_3_	[63,69,88,89,90,91,92,93,94,95,96]
	AgBF_4_	[97]
	AgBF_6_	[97]
	AgClO_4_	[98]
Au	HAuCl_4_·3H_2_O	[69,74,79,85,99,100,101]
	KAuCl_4_	[102,103]
Ru	RuCl_3_·3H_2_O	[75,104,105,106,107]
	Ru(NO)(NO_3_)_3_	[108]
Cu	CuSO_4_·5H_2_O	[109,110,111]
	CuCl_2_·H_2_O	[112,113]
	Cu(NO_3_)_2_·3H_2_O	[114,115]

#### 3.3.2. Solvent

In the context of nanoparticle synthesis, solvents are widely used as a medium for dissolving metal precursors, transferring heat and reactants, and dispersing the resulting nanoparticles. The most commonly used solvents include ethanol, toluene, 1-octadecene, and dimethylformamide. However, current synthesis methods predominantly rely on the use of organic solvents with high toxicity. Thus, it is important to design environmentally friendly alternative solvents to reduce and eliminate environmental risks. Alternative solvents include, e.g., water [111], supercritical fluids [116], and ionic liquids [117,118]. The use of water is beneficial to the environment because it is non-toxic, non-flammable, widely available, and in addition, has a low price with abundant reserves. However, the intensive energy inputs involved in water-using production make its use a challenge in view of the demand for energy-efficient production. The interest in using supercritical solvents, such as supercritical water and supercritical carbon dioxide, has been increasing. At temperatures above the critical point, ordinary solvents transform into supercritical fluids with a much larger void volume and greater compressibility. This endows them with a number of unique physical properties, including density, diffusion coefficient, and thermal conductivity [119]. In contrast, ionic liquids, which consist of charged pairs of inorganic and organic ions, are characterized by their liquid state at room temperature. Due to their very low vapor pressure, ionic liquids are considered promising alternative solvents for replacing toxic volatile organic solvents to reduce environmental risks. A major advantage of using ionic liquids as solvents is that the use of stabilizing agents is generally unnecessary, further simplifying reaction systems and reducing material consumption [119].

#### 3.3.3. Reducer

The selection of a suitable reducing agent is also a key factor since the size, shape, and particle size distribution strongly depend on its nature. The introduction of a reducing agent initiates the reduction in the metal precursor. The reduction in metal salts requires matching the reactivity of the reducing agent to the redox potential of the metal. In addition, it has been proved that if the reaction rate is too high during the synthesis process, a large number of metal nuclei are rapidly formed, and too small particles are formed. On the other hand, particle agglomeration occurs if the reaction rate is too slow [63]. As for the choice of a reducing agent, it is very wide and depends on the specific redox thermodynamics. Additionally, often the choice of the most suitable reducing agent is determined experimentally, and its introduced volume is greater than the stoichiometric requirement [119]. In many cases, the activity of reducing agents is strongly determined by the pH of the solution [40]. The most commonly used reducing agents include [119,120]: ethylene glycol, sodium borohydride, oleyl amine, formaldehyde, carbon oxide(II), hydrazine, ethanol, oxalic acid, hydrogen peroxide, vitamin C, citric acid (Table 3).

Most of the reducing agents used are toxic, requiring significant safety protection during laboratory testing and industrial production. Moreover, as mentioned earlier, in nanoparticle synthesis, the reducing agent is introduced in excess, so as a result, the remaining amount of highly reactive reducing agents in the final products is large. Therefore, the great challenge in nanoparticle synthesis is to reduce and eliminate the use of hazardous reducing agents replaced by environmentally viable alternatives. Because of their non-toxicity, polysaccharides are considered green reducing agents for nanoparticle synthesis. The undoubted advantage of using polysaccharides, in addition to the benefits of green chemistry, is that the hydroxyl groups in the structures of polysaccharides provide them with the ability to reduce metal precursors at the same time as allowing polysaccharides to dissolve in water, further avoiding the use of dangerous organic solvents. In addition, the weak chemical interactions between polysaccharides and nanoparticles ensure that the resulting nanoparticles can be easily separated from the reaction mixtures, making the production more energy-efficient. In some cases, polysaccharides can act as both reducing and capping agents [119]. Many research groups have also successfully used glucose [79,121,122], fructose [79,123], or sucrose [79] as reducing agents in the synthesis of metallic nanoparticles.

**Table 3:** A review of reductants used in the synthesis of Ag and Au nanoparticles.

Reducer	Type of Nanoparticles	References
Glucose	Au	[124]
*Foeniculum vulgare* extract	Au	[125]
*Capsicum annum* extract	Au	[126]
Sodium citrate	Au	[127]
Sodium citrate	Au	[128]
Sodium borohydride	Ag	[129]
Sodium borohydride	Ag	[130]
Glucose		
Formaldehyde		
Glucose	Ag	[131]
Sucrose		
Dextran		
Glucose	Ag	[132]
Dextrin		
Hydrazine	Au	[133]
Citrate	Au	[134]
Ascorbic acid		
Hydrogen peroxide		
Cetyltrimethylammonium chloride	Au	[135]

#### 3.3.4. Stabilizer

Since nanoparticles are essentially finely divided bulk materials, they are usually thermodynamically unstable because of the agglomeration phenomenon. Consequently, they need to be kinetically stabilized, and this is usually realized with a stabilizer. Stabilizing agents are widely used in nanoparticle synthesis not only for their protection against aggregation but also for their ability to limit growth and control the morphology of the products [119,136]. Stabilization is achieved by hydration forces, electrostatic forces, steric forces, or a combination thereof (electrostatic forces). Electrostatic stabilization involves only manipulating the balance between attractive and repulsive forces. The hydrative stabilization mechanism is quite powerful for hydrophilic nanoparticles of solids but is less effective in stabilizing metal particles. Steric and electrosteric stabilization involve the adsorption of surfactant, polymer, or polyelectrolyte molecules onto metal particles, resulting in the control of unbalanced van der Waals attraction forces [64]. The stabilizer is usually introduced during nanoparticle formation. The interaction between the stabilizer and the nanoparticle surface is highly dynamic, and its strength and nature often control the long-term stability of the nanoparticle dispersion. The formation of nanoparticles stabilized by the most common stabilizers (surfactants, polymers and dendrimers, organic ligands) is discussed below, and Table 4 summarizes the types of stabilizers most commonly used in the synthesis of metallic nanoparticles.

**Table 4:** Main categories of frequently used stabilizers, based on [119,120,137,138].

Class	Components
Organic ligands	N-terminated:
	oleyl amine
	octadecylamine
	dodecylamine
	O-terminated:
	oleic acid
	linoleic acid
	P-terminated:
	triphenyl phosphine
	tri-n-octylphosphine
	S-terminated:
	thiols
polymers	polyvinyl pyrrolidone
	polyvinyl alcohol
	polyethylene glycol
	polypropylene glycol
	polyacrylic acid
	polyphenylene oxide
dendrimers	polyamido(amine)
	poly(propyleneimine)
surfactants	hexadecyltrimethylammonium bromide
	tetra-N-alkylammonium halides

**Figure 6:**
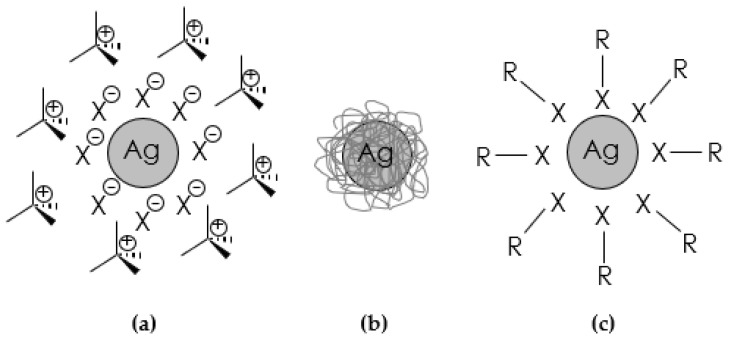
Schematic representation of the ways to stabilize metal nanoparticles (using silver as an example) by using stabilizers: (**a**) surfactants, (**b**) polymers, (**c**) ligands; based on [137].

(a) surfactants—The use of salts/surfactants is a popular route to stabilize metal nanoparticles (Figure 6a). It is believed that surfactant-stabilized nanoparticles strongly adsorb a layer of anions on the metal surface, which in turn are surrounded by a layer (countercations) to maintain electrical neutrality. Both surfactant components play a key role in protecting the metal from agglomeration. Varying the nature of the cationic component allows nanoparticles to disperse in organic or aqueous environments. As far as the ionic surfactants are concerned, a typical approach is to match the opposite charges of metal ions and surfactant ions to perform chemical reduction [137,139]. The choice of surfactant is a key point in all chemical synthesis methods, as its molecular structure, its concentration, and even its mixing time in the reaction medium have a fundamental impact on the kinetics and geometry of metallic nanophases. Non-ionic surfactants are characterized by the absence of dissociative groups on the hydrophilic part of their molecules, such as ethoxylated alcohols, ethoxylated amines, amine oxides, and thiols. They are less sensitive to electrolytes than ionic surfactants, so they can be used even at high concentrations of dissolved salts. Ionic surfactants, on the other hand, are grouped into anionic and cationic types according to the sign of the charges located on the hydrophilic part of their molecule. Alkyl sulfates, and alkylammonium salts are the most common anionic and cationic surfactants used in the synthesis of metal nanoparticles, respectively. In both cases, their hydrophilicity is controlled by changing the length of the alkyl chain [39].

(b) steric stabilizers (polymers and dendrimers)—stabilization of nanosystems can also be achieved by incorporating them into an organic matrix, which can be either a flexible polymer or a more organized dendritic structure (Figure 6b). The steric weight of this class of stabilizing agents prevents the agglomeration of nanoparticles. Polymers provide stabilization of nanoparticles through their binding affinity to the surface and also through the spatial mass of their three-dimensional structure [119]. Dendrimers are hyperbranched macromolecules with a high concentration of functional groups whose cavities behave like molecular boxes that can trap and stabilize metal nanoparticles, especially if heteroatoms are inside the dendrimer. Dendrimers retain the guest molecule through covalent bonding, electrostatic forces, or van der Waals forces [140]. Polymers such as polyvinyl pyrrolidone and polyvinyl alcohol are widely used to protect nanoparticles because of their commercial availability at relatively low cost and their solubility in a range of solvents, including water [137,139]. The main advantages of using dendrimers include:

- the possibility of obtaining monodisperse nanoparticles due to the high homogeneity and porosity of the dendrimers,
- prevention of nanoparticle agglomeration due to the steric effect of the dendrimers,
- possibility of application in catalysis, among other things, since the nanoparticles are only partially surrounded by the dendrimer,
- acting as a “nanofilter” to control access of small molecules to the attached nanoparticles (depending on types of functional groups and solvents),
- the possibility of changing the solubility between the hydrophilic dendrimer and hydrophobic metal molecules due to the fact that the end groups of the dendrimer can be combined with other functional groups.

(c) organic ligands—one of the most common methods of stabilizing metal nanoparticles is the addition of an organic ligand, which usually contains a heteroatom with a free electron pair (Figure 6c). The organic chain of the ligand prevents agglomeration, while the heteroatom binds strongly to the metal surface. The most commonly used ligands include those based on sulfur, phosphorus, oxygen, and nitrogen [137].

In the literature on the synthesis of metallic nanoparticles, one can increasingly often encounter the use of naturally occurring chemicals as stabilizing agents. As already mentioned when describing alternative reducing agents, polysaccharides can act as reducing agents but also as stabilizers. It is not surprising, for with the growing efforts to minimize or completely eliminate waste and implement sustainable processes by adopting the 12 basic principles of Green Chemistry, it is desirable to develop biological and biomimetic approaches to the preparation of advanced materials. Most commonly, chitosan [141,142], starch [143], heparin [144,145], and also chemical compounds belonging to the group of biomolecules are used as natural stabilizing agents.

### 3.4. Characterization of Metallic Nanoparticles

Nanostructures have attracted tremendous interest as a rapidly growing class of materials suitable for many applications. Numerous techniques exist to characterize the size, crystal structure, elemental composition, and a range of other physical properties of nanoparticles. Certain physical properties can be assessed by more than one technique. Different advantages and limitations of each technique complicate the selection of the most appropriate method, and in fact, quite often, a broader characterization of nanoparticles is necessary, requiring a comprehensive approach by combining techniques in a complementary manner. In this context, it is desirable to know the limitations and advantages of different techniques in order to know whether, in some cases, the use of only one or two of them is sufficient to provide reliable information when studying a specific parameter [146]. It is worth mentioning that the characterization of nanoparticles is carried out using various techniques, mainly taken from materials engineering—most of these techniques are summarized in Table 5 [5].

The physicochemical properties of nanomaterials largely depend on their three-dimensional morphology (size, shape, and surface topography), the surrounding media, and their distribution in space. The correlation of these parameters with the corresponding physical and chemical properties is a fundamental requirement for discovering new properties and applications, as well as for advancing the fundamental and practical knowledge required to design and manufacture new materials [35]. In order to characterize nanoparticles, it is useful to first find answers to the three fundamental questions [147]:

- What does the material look like (size, size distribution, shape, topography, degree of agglomeration, aggregation)?
- What is the material made of (chemical composition, crystal structure, purity, impurity level, elemental composition, chemical composition, and phase composition)?
- Which factors affect the material’s interaction with its environment (specific surface area, surface chemistry, surface charge)?

**Figure 7:**
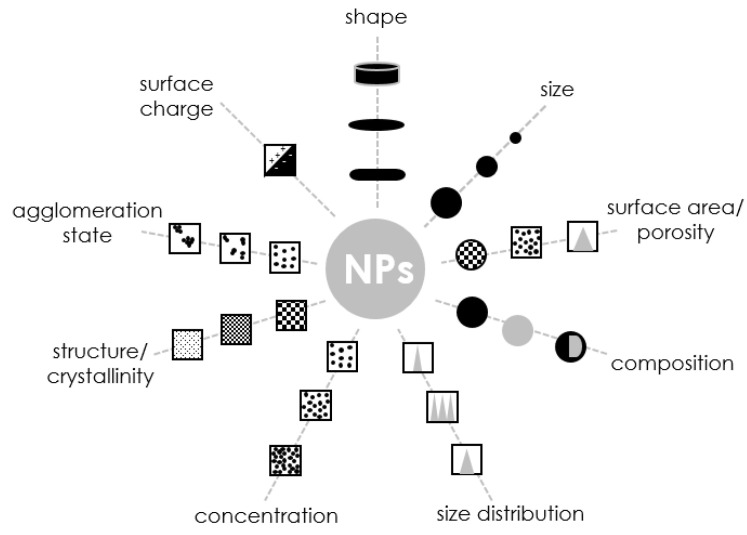
Schematic representation of parameters needed to be determined to characterize nanoparticles, based on [147].

Although there are many different information needs for nanoparticles and other nanostructured materials, considerable efforts have been made to identify the most necessary or required property measurements, which are shown in Figure 7. Although the determination of the size, distribution, and particle shape is vital, the surface properties of nanoparticles are also important (specific surface area, surface charge, and degree of agglomeration) [147]. Since the proportion of atoms and molecules near the surface increases significantly for nanostructured materials, it is not surprising that surface properties play a significant role in determining the behavior of such materials. The most important parameters that provide sufficient information about the nanoparticles in question are given below.

#### 3.4.1. Size

As mentioned on the first pages of this paper, nanoscale objects are all materials with at least one dimension smaller than 100 nm. The size of nanoparticles is one of the most important factors affecting their physicochemical and functional properties [148]. Thus, it should be the first parameter to be measured when characterizing these materials. Size refers to the spatial extent of an object. For a spherical object, it can be unambiguously described by a single dimension, but since many particle systems have an irregular shape, usually, the particle size is expressed as an equivalent spherical diameter [149]. For non-spherical objects, several dimensions are needed to fully define the actual extension of the object in space. While measuring size may seem trivial for macroscopic-scale objects, at the nanoscale, it takes on different meanings according to the technique used to measure it. In the case of nanoparticles, size can refer to (i) its overall physical dimension defined by its atomic structure, (ii) the effective size of a particle in a specific matrix according to its diffusion/sedimentation behavior, (iii) the effective size of a nanoparticle-based on its mass/electron distribution. This variety of size definitions reflects the broad spectrum of physical approaches that can be used to characterize nanoparticles [150]. In principle, there is a variety of analytical instruments that can be used to measure the size of nanoparticles; however, many require specialized equipment that is not widely available for general use [148]. Some of the simplest techniques with which to measure the size and morphology of nanoparticles at the single particle level at sub-nanometer resolution are high-resolution microscopy techniques. Methods based on light scattering, diffusion, and sedimentation are commonly used to analyze colloidal suspensions. However, it is usually not possible to obtain direct information about the shape of nanoparticles from these approaches, and the equivalent diameter corresponding to that of a sphere behaving in the same way as the sample under study is usually referred to as the characteristic size. To translate this information into the actual dimensions of NPs, knowledge of their shape is required. Finally, static scattering methods, using light or X-rays, provide information about the mass/electron distribution of nanoparticles and, therefore, indirectly about their shape [150].

#### 3.4.2. Size Distribution

Typically, the goal of nanoparticle synthesis is to obtain a monodisperse population; however, it should be kept in mind that the actual sample always exhibits a certain degree of variability. Therefore, the size distribution of nanoparticles is an intrinsic measure of the control and quality of the synthesis procedure, while the estimated size value refers only to the averaged amount obtained from this distribution. There are many techniques for measuring particle size distribution based on a variety of physical principles; some examples include laser diffraction, dynamic light scattering, microscopy, and surface area measurements [149]. Typically, the size properties of nanoparticles are represented by the particle size distribution in combination with measures of central tendency (such as mean diameter) and width of distribution (such as polydispersity index). When reporting particle size data, it is always important to specify whether the particle dimensions are given as radius or diameter [148].

#### 3.4.3. Shape

Although particles are often assumed to be spherical, they actually have a wide variety of geometric shapes and can also take on spherical, planar, cylindrical, tubular, or conical shapes. Nanoparticles can also have complex internal structures, such as homogeneous or core-shell. They may occur as isolated particles or may be organized into different types of structural arrangements, such as chains or flocs. The morphology and organization of nanoparticles play an important role in determining their physicochemical and functional properties, so it is important to know analytical methods for measuring these properties [148]. It has been proven that particles with the same composition and similar dimensions can exhibit drastically different behavior due to their different shape. The shape is usually characterized using high-resolution microscopy techniques, which allow the morphology of sub-nanometer particles to be determined. However, electron microscopy typically provides a 2D projection of a particle’s shape onto a plane, which in the special cases of highly anisotropic particles, can lead to erroneous estimates of particle morphology. To circumvent this limitation, the characterization of particles exhibiting pronounced 3D anisotropy can be performed by acquiring projections of a large number of randomly oriented identical particles to reconstruct their spatial arrangement or electron tomography. Information on particle shape and anisotropy can also be obtained by using scattering-based techniques that can be more easily applied in solution, for example, by combining static and dynamic light scattering techniques. However, such particle analysis only allows quantitative inference of the particle’s anisotropy coefficient, and detailed study of particle morphology remains limited to high-resolution microscopy. Nevertheless, qualitative shape information obtained by scattering-based characterization methods is often necessary to confirm microscopy results since sample preparation and evaluation by electron microscopy can affect the agglomeration state of the sample or induce damage to the particle framework [150].

#### 3.4.4. Surface Charge

The surface charge of nanoparticles is important because it determines their interaction with other charged particles in their environment. The boundary between the solid and liquid phases is a dynamic environment, and numerous phenomena contribute to the appearance of charge on the surface of nanoparticles [150], including the presence of ionized components on their surfaces, such as ionic surfactants, phospholipids, proteins or polysaccharides [148]. This charge not only influences the behavior of nanoparticles in different environments but also controls their tendency to aggregate since electrostatic repulsion between molecules is a key factor promoting the stability of colloidal solutions. In particular, in an electrolyte solution, mobile charges are attracted by static charges on the surface of nanoparticles, effectively leading to electric potential shielding, which can eventually lead to particle aggregation [150]. The electrical characteristics of nanoparticles depend on the type, concentration, and location of functional groups on their surface, as well as the ionic composition and physical properties of the surrounding liquid [148]. A typical measure of surface charge and colloidal stability is the zeta potential. Several parameters affect the zeta potential of particles in solution, namely the ionic strength of the solvent, the presence of charged or uncharged particles that can adsorb to the particle surface, and the pH of the solution [150]. The electrical properties of nanoparticles are also often characterized by surface charge density and surface electric potential [148]. To sum up, the electrical charge of nanoparticles is a key characteristic that is extremely important in determining colloidal stability, particle self-organization, chemical catalysis, and various biomedical applications [151].

#### 3.4.5. Surface AREA/porosity

Surface area is another significant factor in characterizing nanoparticles. The surface area-to-volume ratio of a nanoparticle has a huge impact on its performance and properties. The ability to synthesize nanoparticles containing porous frameworks has greatly expanded the application range of nanomaterials. Porosity provides nanoparticles with a dramatically increased surface-to-volume ratio, which can exceed that of solid particles of equal size by several orders of magnitude. To permit the development and characterization of porous nanoparticles, it is necessary to study porosity at different levels, namely (i) the size of the pore opening, (ii) the dimensions and volume of the porous cavity, (iii) the connectivity of the porous structure, (iv) the specific surface area (the sum of the inner and outer surfaces), (v) the surface-to-volume ratio, and (vi) the functionalization of the inner and outer surfaces [150]. The most commonly used and one of the simplest methods for measuring surface area is BET analysis.

#### 3.4.6. Concentration

The total concentration of nanoparticles present in a sample after they have been manufactured is a very important issue. Sometimes the total concentration of nanoparticles is known due to the nature of the ingredients and the processing used to make them, but in other cases, it may be necessary to measure the concentration of particles, so appropriate analytical methods are needed. Many analytical methods are available for determining the total concentration of particles in a suspension. If there are no significant levels of solutes in the surrounding liquid, the nanoparticle suspension can simply be dried and weighed. If solutes are present, the nanoparticles can be separated by centrifugation, dialysis, filtration, or selective precipitation, then dried and weighed. If the nanoparticles scatter light significantly, then the concentration can be determined by simply measuring the turbidity after establishing an appropriate turbidity calibration curve as a function of particle concentration. The main limitation of this method is that the turbidity of a colloidal dispersion depends on the size of the particles as well as their concentration. This method may therefore be most suitable when the size of the nanoparticles is already known or can be measured independently. In addition, many nanoparticles may only scatter light weakly due to their small size relative to the wavelength of light, and thus, may not cause a large change in turbidity with particle concentration [148].

#### 3.4.7. Composition

The most appropriate method for determining the composition of nanoparticles depends on their exact nature, i.e., the type and amount of their various components [148]. In principle, determination of the purity of nanomaterials can be achieved by analyzing their chemical composition, for it is the chemical or elemental composition that determines the purity and, therefore, the performance of nanoparticles. The presence of higher secondary or undesirable elements in a nanoparticle can reduce its performance and lead to secondary reaction and contamination. Analysis of the chemical composition of nanomaterials is more complicated than that of a single unit [152]. Among a wide variety of methods, the characterization of nanomaterial’s composition is usually carried out by X-ray photoelectron spectroscopy [148].

#### 3.4.8. Agglomeration State

The dispersion state of a particulate system describes the degree of agglomeration of particles that coexist in groups or clusters through the action of various inter-particle forces, the most fundamental of which are van der Waals forces. The magnitude of these forces is a function of the fundamental atomic and molecular properties of the surface atoms, the surface morphology, and the proximity of these surfaces [149]. For most measurement techniques, the size distribution of existing agglomerates is quantified. Thus, the measured size distribution is highly dependent on the dispersion state of the system. Due to attractive forces, particles will tend to agglomerate in suspension unless they are stabilized by equivalent repulsive forces, such as surface charge or steric effects. However, the smaller the particle size, the greater the relative attractive forces per unit mass. This means that once agglomerated, it becomes increasingly difficult to disperse materials at the nanoscale as the size decreases [149]. The dispersion of nanoparticles in solution is usually measured using dynamic light scattering, as this technique provides measurements of a large number of particles in solution and provides a robust and quantitative assessment of narrow size distributions. A technique that complements dynamic light scattering is transmission electron microscopy which is mainly used to measure the primary size of nanoparticles [153].

### 3.5. Examples from Literature

As has been mentioned several times, the method for the synthesis of metal nanoparticles by chemical reduction allows the production of nanomaterials with controlled morphology. To illustrate how changing various synthesis parameters affects the final nanoparticles, selected examples from the literature are described below.

Researchers from the Suriati research group [63] have synthesized silver nanoparticles by chemical reduction, which turned out to be a simple, inexpensive, and partly green method as they used ascorbic acid (C_6_H_8_O_6_), which is one form of vitamin C, as a surfactant. The metal precursor was silver nitrate (AgNO_3_), while the reducing agent was sodium citrate (C_6_H_5_O_7_Na_3_). The concentrations of sodium citrate and ascorbic acid were varied to observe the effects of these parameters, particularly on the size and morphology of the silver nanoparticles.

- The effect of the reducer concentration

At the reducing agent concentrations of 4.0–8.0 mM, all the nanoparticles produced had a quasi-spherical shape. In contrast, it was observed that the nanoparticle sizes showed a decreasing trend with increasing concentration of the reducer, from 38.53 nm at 4.0 mM C_6_H_5_O_7_Na_3_ to 36.32 at 8.0 mM C_6_H_5_O_7_Na_3_. Moreover, with increasing concentration of the reducer, a narrowing of the particle size distribution was observed from 20–65 nm to 20–50 nm. A possible reason for this phenomenon could be that the rate of the reaction is directly proportional to the concentration of the reactant according to the law of action of masses, from which it follows that as the concentration of trisodium citrate increased, the rate of the reaction increased. It has been concluded that as the reaction rate increased, the silver ions were consumed faster, leaving less room for particle size growth.

- The effect of the stabilizer concentration

The TEM observations have shown that the average size of silver nanoparticles increased as the concentration of ascorbic acid increased from 1.0 mM to 4.0 mM. The average sizes of silver nanoparticles produced at 1.0 mM, 2.0 mM, 3.0 mM, and 4.0 mM ascorbic acid were 37.24 nm, 43.04 nm, 45.85 nm, and 47.28 nm, respectively. It seemed likely that ascorbic acid was able to kinetically control the growth rate of various surfaces by selectively adsorbing on these surfaces. Because of the acidic properties of ascorbic acid, the addition of a high concentration of ascorbic acid subsequently lowered the pH of the solution.

The work reported by Chou and co-workers [94] investigated the synthesis of silver nanoparticles by the chemical reduction method using silver nitrate (AgNO_3_) as a metal precursor, formaldehyde (CH_2_O) as a reducing agent, and polyvinylpyrrolidone/poly(vinyl alcohol), (PVP/PVA) as stabilizing agents. A solution of either sodium carbonate (Na_2_CO_3_) or sodium hydroxide (NaOH) was used to determine the preferred pH. The effect of the amount of alkaline solution on the morphology of the final nanoparticles was examined.

- The effect of pH

**Figure 8:**
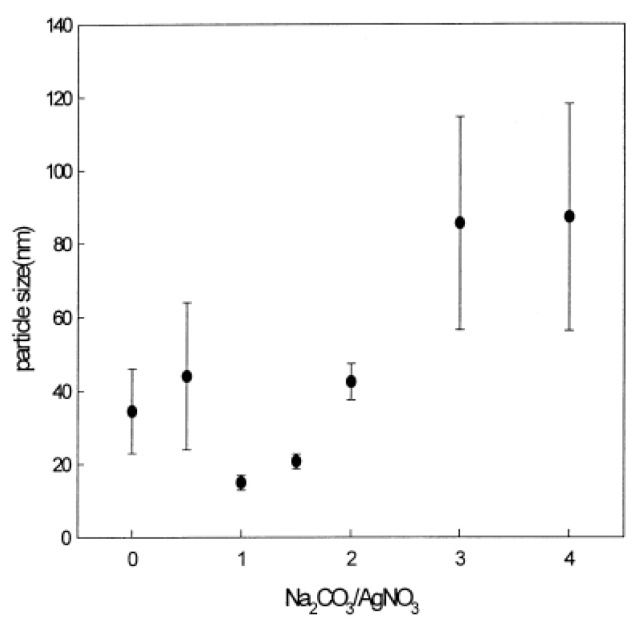
Effect of Na_2_CO_3_/AgNO_3_ ratio on the silver average size and its standard deviation. Reproduced with permission from [94].

Although high pH is preferred in nanoparticle synthesis due to its higher reducing power, Chou’s research group has shown an adverse effect of high pH on particle size. When more NaOH was added to the reaction system, the silver colloids settled at the bottom of the solution. Thus, NaOH was replaced with a weak base, Na_2_CO_3,_ in order to release hydroxyl ions only when the pH fell below certain values. The effect of the amount of sodium carbonate on the average particle size and also the standard deviation of the particle size distribution is shown in Figure 8. As shown in this figure, the optimal conditions were at a ratio of Na_2_CO_3_/AgNO_3_ between 1.0 and 1.5, at which smaller nanoparticle sizes were obtained. When more Na_2_CO_3_ was added, the pH of the solution increased, which adversely affected the stability of the silver colloids; moreover, the size of the nanoparticles increased from 10–20 nm to 80–100 nm, as did the size distribution, as indicated by the bar shown in the figure below.

Liguo’s research group [95] prepared silver nanoparticles by the chemical reduction method, using hexadecyltrimethylammonium bromide (CTAB) to prevent nanoparticle agglomeration, silver nitrate (AgNO_3_) as the metal source and formaldehyde (CH_2_O) as the reducing agent. The pH of the solution was adjusted by adding nitric acid (V) (HNO_3_) or sodium hydroxide (NaOH) to the solution mixture at the nanoparticle synthesis stage.

- The effect of the stabilizer concentration

**Figure 9:**
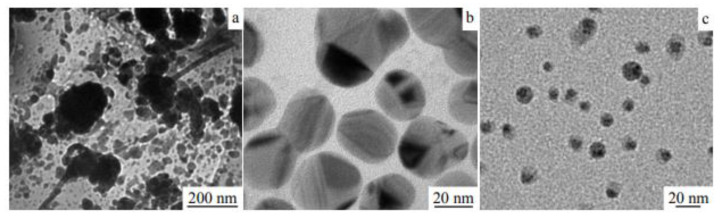
TEM morphologies of nano-silver particles with different CTAB amounts, CTAB/AgNO_3_: (**a**) 0.5, (**b**) 0.8, (**c**) 1.2. Reproduced with permission from [95].

The addition of a small amount of CTAB to the solution resulted in the formation of particles of large size, irregular shape, and significant agglomeration degree (Figure 9a). As the amount of CTAB increased, the particle sizes were better dispersed. When the CTAB/AgNO_3_ ratio was 0.8, nanosilver particles of 20–40 nm were obtained (Figure 9b), while when this ratio reached 1.2, then particles smaller than 10 nm were formed (Figure 9c).

It has been shown that CTAB has an inhibitory effect on the process of particle growth and agglomeration, which was explained by the effect of long carbon chains of CTAB, which may reduce the possibility of collisions between silver particles. In addition, CTAB, due to its structure, exhibits a strong steric effect. Thus, at low concentrations, it had little effect on the particle size and agglomeration, and as its concentration increased, better dispersion was achieved. On the other hand, an excessive amount of CTAB retards the growth of particles, as they are wrapped inside CTAB molecules, which prevents the growth of nuclei resulting in the formation of particles of several nanometers in size.

- The effect of temperature

**Figure 10:**
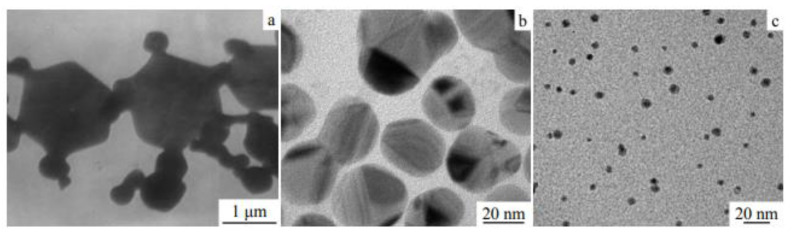
TEM morphologies of nano-silver particles at different reaction temperatures: (**a**) 20 °C, (**b**) 40 °C, (**c**) 60 °C. Reproduced with permission from [95].

As established by Liguo et al., the reaction temperature affects the morphology of silver particles in two ways. On the one hand, the rate of reduction in silver nitrate depends on temperature, while on the other hand, temperature affects the interactions, in this case, between CTAB and Ag^+^. As a result, both temperature-dependent processes ultimately affect the morphology of the nanosilver particles. The morphological response of particles prepared at different temperatures was examined. TEM images of the obtained nanoparticles at the reaction temperatures of 20 °C, 40 °C, and 60 °C are presented in Figure 10a, b, and c, respectively. When the reaction temperature was 20 °C, heterogeneous particles of about 100 nm in size were obtained. With increasing reaction temperature, the particles became smaller—their size was close to 30 nm at 40 °C, while at 60 °C, it was about 10 nm.

These observations were interpreted as follows. When the reaction temperature is low (20 °C), CTAB dissolves poorly, and in addition, precipitation is often easier, which ultimately leads to a small amount of CTAB participating in the reaction, further resulting in a reduced effect on inhibiting particle growth. On the other hand, the reducing ability of formaldehyde is very weak at low temperatures. As a result, the reaction is slow, and the initial nuclei can then consume most of the reduced silver atoms. The number of further forming nuclei is smaller and large particles with a wide size distribution are eventually formed. At 40 °C, CTAB is fully dissolved, and the amount of silver nitrate decreases at a moderate rate. The nanosilver particles obtained exhibit a flake morphology. When the temperature reaches 60 °C, the reduction in silver cations is faster. A large number of silver atoms are generated in a short time, and the nucleation rate is greatly increased. The nucleation process consumes most of the silver reserves, which inhibits the growth of newly formed particles, so small particles are formed.

- The effect of pH

**Figure 11:**
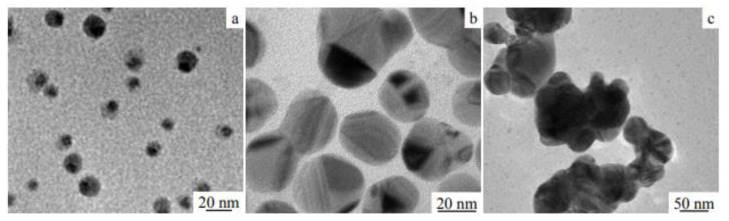
TEM images of nano-silver particles under different pH conditions: (**a**) pH = 3, (**b**) pH = 6, (**c**) pH = 9. Reproduced with permission from [95].

The morphology of silver nanoparticles at different pH was also examined. As shown in Figure 11, at pH of 3, the particle size was less than 20 nm; at pH increased to 9, the agglomeration of the particles formed was advanced; while at pH of 6, the particles showed a flaky structure and good dispersion. The explanation proposed was that at a low pH value, the reduction ability of formaldehyde is weak, resulting in a low reaction rate. On the other hand, the presence of a large number of H^+^ in the solution inhibits the reaction and causes an incomplete reaction of silver cations.

In addition, after the reactions were complete, the contents of Ag^+^ with Cl^−^ at different pH were checked. At pH of 3, the reduction in Ag^+^ was incomplete because fewer silver atoms existing in the solution participated in the nucleation and particle growth processes, eventually leading to smaller nanosilver particle formation. At weakly acidic pH, a small amount of H^+^ in solution is beneficial for maintaining the stability of the bilayer surface, which favors the formation of stable nanoparticles with good dispersion.

Guzmán and co-workers [91] have synthesized silver nanoparticles by the chemical reduction method using silver nitrate (AgNO_3_) as the metal precursor, hydrazine hydrate (N_2_H_4_), and/or sodium citrate (Na_3_C_6_H_5_O_7_) as the reducing agent and sodium dodecyl sulfate (SDS) and/or sodium citrate (Na_3_C_6_H_5_O_7_) for stabilizing the whole system. The effect of the concentration of sodium citrate and the type of reducing agent used on the morphology of the final particles was investigated.

- The effect of the reducer concentration

**Figure 12:**
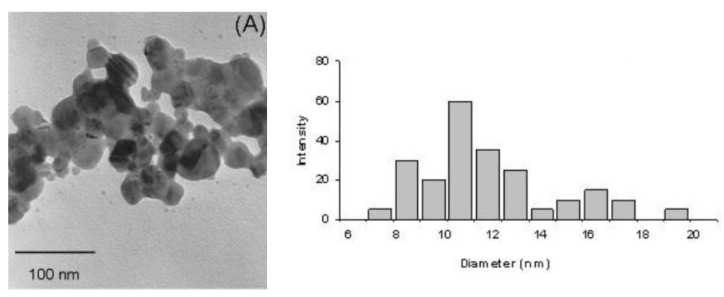

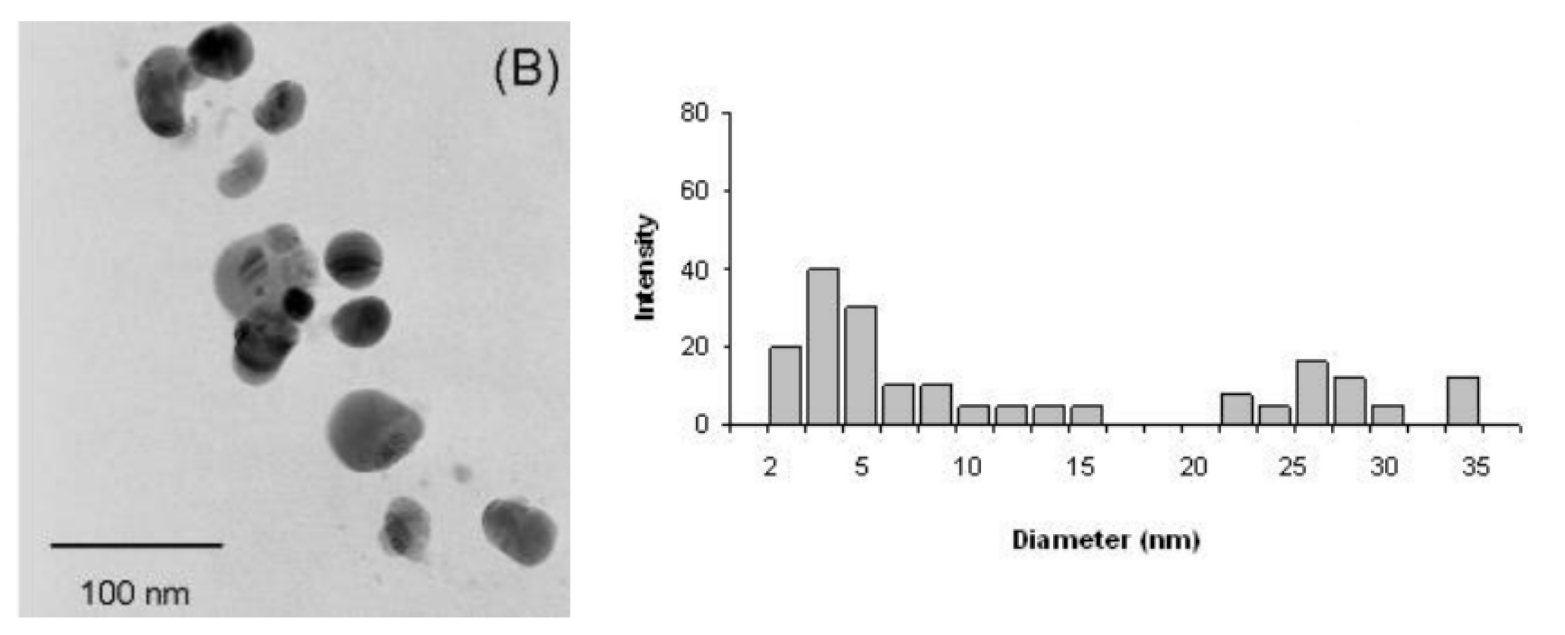
TEM image and particle size distribution of silver nanoparticles obtained at 1.0 mM (**A**) and 2.0 mM (**B**) of citrate of sodium solution. Reproduced with permission from [91].

TEM studies indicated a correlation between the concentration of sodium citrate and the morphology of the final nanoparticles. For hydrazine at a concentration of 2.0 mM and sodium citrate at 1.0 mM, the silver nanoparticles were obtained in the form of small, significantly agglomerated grains (Figure 12A, left). In addition, the obtained particle size histogram showed the size range of the silver nanoparticles from 7 to 20 nm with an average diameter of 9 nm (Figure 12A, right). In contrast, the nanoparticles obtained at twice the concentration of sodium citrate and the same concentration of hydrazine assumed a spherical shape and were additionally characterized by good dispersion (Figure 12B, left). From the histogram, it was deduced that, in this case, the silver particles assumed sizes ranging from 7 to 20 nm and 22 to 35 nm, with an average diameter of 11 nm (Figure 12B, right). The described relationship was also confirmed by UV-Vis studies, which revealed typical plasmonic absorption maxima at 405 nm and 406 nm when the sodium citrate solution of 1.0 mM and 2.0 mM, respectively, was used. The observed differences in the position and shape of plasmonic absorption are due to differences in the particle size, shape, and dielectric constant of the surrounding medium.

- The effect of the type of reducer

In addition, the effect of the type of reducing agent on the particle size was examined. When hydrazine was used as a reducing agent, the average diameter of the particles obtained was about 30 nm. On the other hand, when a mixture of hydrazine and sodium citrate was used as a reducing agent, the average particle diameter was in the range of 15–48 nm, which, compared to the particle size obtained with hydrazine alone, indicates a slight increase in the average particle diameter.

Song and co-workers [89] have synthesized silver nanoparticles using silver nitrate (AgNO_3_) as the precursor while sodium borohydride (NaBH_4_) and sodium dodecyl sulfate (SDS) as the reducing agent and stabilizing agents, respectively. The effects of several variables, i.e., the concentration of AgNO_3_, NaBH_4,_ and SDS, on the final silver nanoparticles were examined.

- The effect of metal precursor concentration

**Figure 13:**
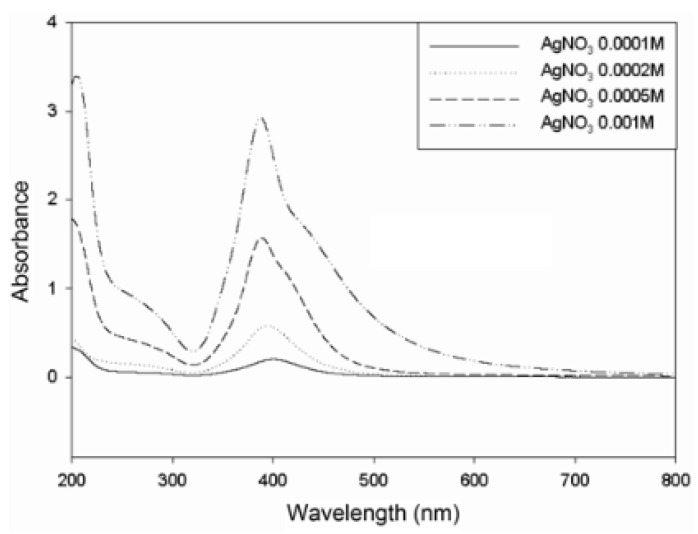
UV-Vis absorption spectra of the silver nanoparticles prepared via reduction in AgNO_3_ at different initial concentrations. Reproduced with permission from [89].

Figure 13 shows the UV-Vis spectra of colloidal silver nanoparticles prepared at different initial concentrations of AgNO_3_ (0.0001 M, 0.0002 M, 0.0005 M, and 0.001 M). The nanoparticles were synthesized at the NaBH_4_/AgNO_3_ molar ratio of 10 and SDS/AgNO_3_ weight ratio of 2. The color of the solutions depended on the concentration of AgNO_3_ added. As the initial concentration of AgNO_3_ increased, the color of the solution changed from yellow to brown. The absorption peak at about 400 nm was attributed to plasmonic excitation by silver nanospheres, indicating the formation of silver nanoparticles. At low concentrations of AgNO_3_, the maximum weak absorption of surface plasmon peaks was observed at 400 nm, indicating that silver nanoparticles were produced at relatively low concentrations. As the AgNO_3_ concentration increased, the intensity of the maximum plasmonic peak increased, indicating that higher concentrations of silver nanoparticles were formed.

- The effect of reducer concentration

**Figure 14:**
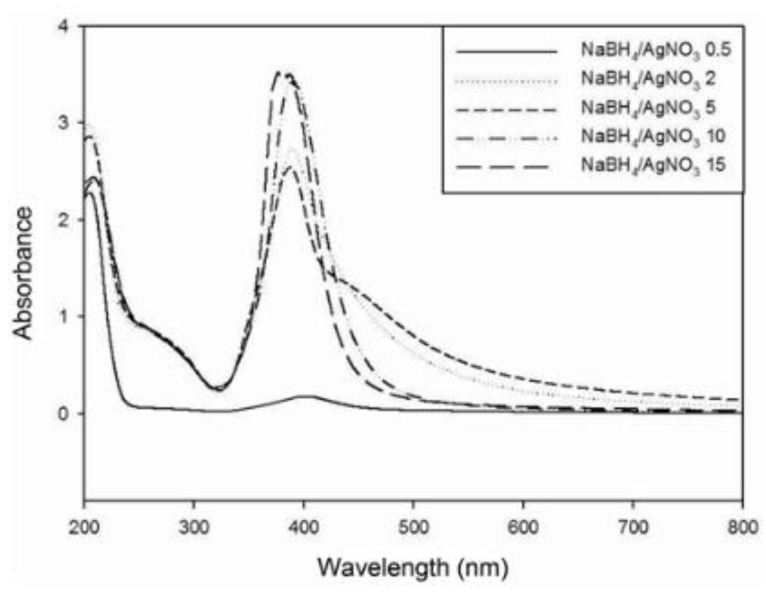
UV-Vis absorption spectra of the silver nanoparticles prepared at different NaBH_4_/AgNO_3_ molar ratios. Reproduced with permission from [89].

To understand the effect of NaBH_4_ concentration, the reduction reaction was studied at different NaBH_4_/AgNO_3_ molar ratios (0.5–15), at the initial AgNO_3_ concentration of 0.001 M, and the SDS/AgNO_3_ weight ratio of 2. The corresponding UV-Vis spectra are shown in Figure 14. At the lowest NaBH_4_/AgNO_3_ molar ratio, a weak plasmonic peak was observed at 400 nm, indicating a relatively low concentration of silver nanoparticles formed, the reason being insufficient reduction. It is known that the UV-Vis absorption peak can also provide information about the degree of dispersion of silver nanoparticles. The narrower it is, the better the dispersion degree of nanoparticles is obtained. At molar ratios of 2 and 5, the absorption peak at 400 nm was broad, indicating that the silver nanoparticles were aggregated, while when the molar ratios were 10 and 15, narrow absorption peaks were obtained, indicating that the silver nanoparticles were well dispersed. The reason for this, according to Song et al., was the use of too little NaBH_4_ so that boron hydroxide B(OH)_3_ (produced by hydrolysis of NaBH_4_, see Equation (2)) was absorbed into the silver nanoparticles, reducing the electron density and causing deep aggregation. On the other hand, when an excessive amount of NaBH_4_ was used, a thick layer of BH^4−^ prevented the absorption of boron hydroxide onto the surfaces of silver nanoparticles, resulting in well-dispersed nanoparticles. These results indicate that NaBH_4_ acted not only as a reducing agent but also as a stabilizer protecting against the aggregation of silver nanoparticles.

Ag^+^ + BH^4−^ + 3H_2_O → Ag^0^ + B(OH)_3_ + 3.5H_2_ (2)

- The effect of stabilizer concentration

**Figure 15:**
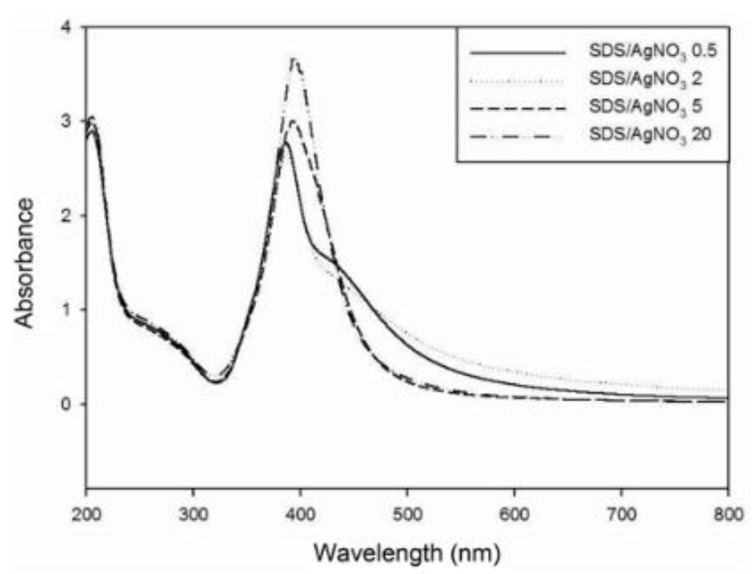
UV-Vis absorption spectra of the silver nanoparticles prepared with different SDS/AgNO_3_ weight ratios. Reproduced with permission from [89].

The main purpose of introducing SDS into the solution was to prevent the growth and aggregation of silver nanoparticles. Figure 15 shows UV-Vis spectra of silver nanoparticles with different SDS/AgNO_3_ weight ratios (0.5–20). The nanoparticles were synthesized under conditions of an initial AgNO_3_ concentration (0.001 M) and a NaBH_4_/AgNO_3_ molar ratio of 4. As the SDS concentration increased, the color of the solutions changed from brown to yellow. At high SDS/AgNO_3_ weight ratios (5, 20), narrow plasmonic absorption peaks were observed at 400 nm, confirming the nanocrystalline nature and well-dispersed state of the silver particles. However, when the weight ratios were low (0.5, 2), the absorption peaks became broad, indicating that the silver nanoparticles were aggregated. These results imply that with the right amount of SDS, it absorbs the surface of silver nanoparticles and protects them from steric growth and aggregation.

Researchers from the Alqadi research group [96] synthesized silver nanoparticles, where they controlled their size by changing the pH value of the reaction system. For the synthesis, they used silver nitrate (AgNO_3_), which was reduced with ascorbic acid (C_6_H_8_O_6_), while sodium citrate (Na_3_C_6_H_5_O_7_) was used as a stabilizer. The pH was manipulated with the addition of either sodium hydroxide (NaOH) or citric acid (C_6_H_8_O_7_).

- The effect of pH

**Figure 16:**
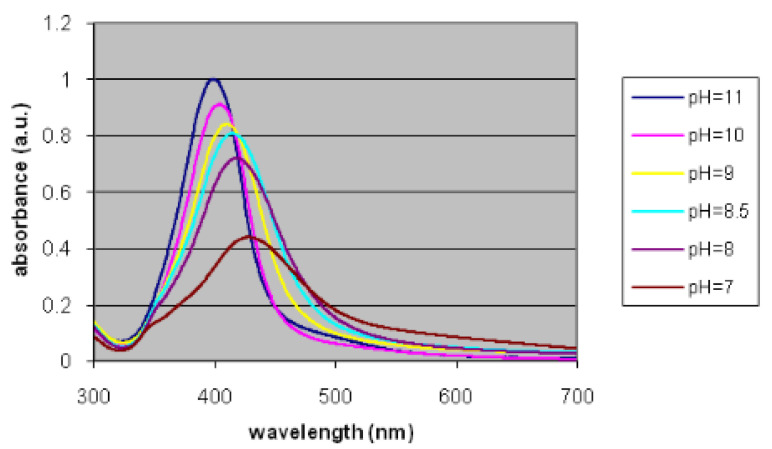
Absorption spectra of AgNPs at different pH values. Reproduced with permission from [96].

The changes in the size of silver nanoparticles in response to a change in the pH of the solution were monitored. Absorption spectra recorded at different pH values are shown in Figure 16. Based on the plasmon resonance peaks obtained, it was found that at high pH, silver nanoparticles of smaller sizes were obtained (the plasmon resonance peak shifted towards the short wavelength region as well as increasingly narrow peaks) compared to those obtained at low pH values. The difference was attributed to the different rates of precursor reduction.

**Figure 17:**
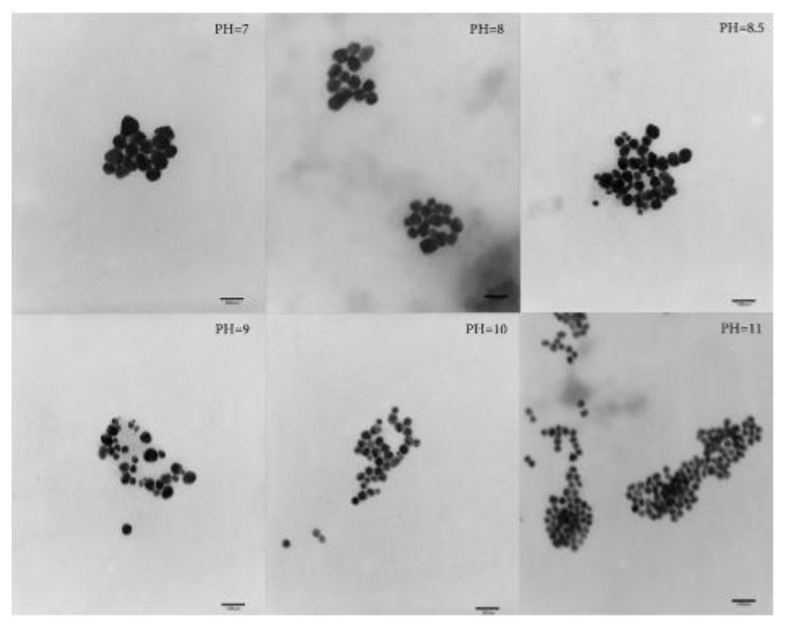
TEM image of nanoparticles formed at different pH values. Reproduced with permission from [96].

In addition to the inverse proportionality between particle size and pH value, it is clear that increasing the pH value yields spherical nanoparticles, while at low pH, rods and triangular particle shapes were formed, as shown in Figure 17. The irregularity of the particle shape was attributed to the slow reduction rate of the precursor, as well as the poor balance between nucleation and growth processes.

Beyribey and co-workers [83] have synthesized platinum nanoparticles by the chemical reduction in hexachloroplatinic acid (H_2_PtCl_6_) with hydrazine (N_2_H_4_). The purpose of the experiment was to study the effect of temperature and pH on the structure of platinum particles.

- The effect of temperature and pH

**Figure 18:**
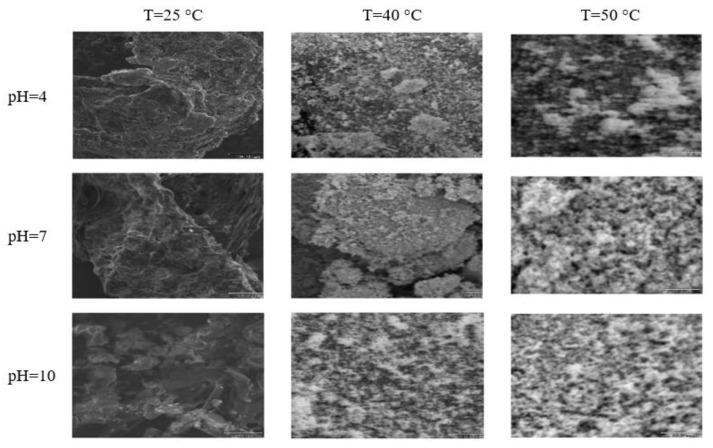
SEM micrographs at different temperatures and pH values. Reproduced with permission from [83].

The synthesis of platinum nanoparticles was carried out at pH equal to 4, 7, or 10 and at 25 °C, 40 °C, or 50 °C. At the low pH of the solution, no characteristic structures of platinum nanoparticles formed; they were only observed when the pH of the solution was 10. A significant difference in the morphological distribution of platinum particles at high temperatures and pH was also observed compared to that of the nanoparticles obtained at lower temperatures and lower pH values, as shown in the photographs below (Figure 18).

The zeta potential, informing about the physical stability of emulsions and suspensions, was also measured. If all the particles in a suspension have a high negative or positive zeta potential, then they will repel each other and will not tend to flocculate. However, if the particles have low zeta potential values, then there is no force to prevent the particles from clumping together. The general dividing line between stable and unstable suspensions is usually taken at +30 mV or −30 mV. Particles with a zeta potential more positive than +30 mV or more negative than −30 mV are usually considered stable. It has been shown that platinum particles synthesized at 25 °C in the pH range of 4–7 are unstable (Table 6).

An equally interesting study was conducted by scientists from the Patharkar research group [105], who synthesized ruthenium nanoparticles by the chemical reduction in ruthenium chloride (RuCl_3_) using sodium borohydride (NaBH_4_) as a reducing agent and sodium dodecyl sulfate (SDS) as a stabilizer (other stabilizers such as PVP, CTAB, and AOT were also used). The influence of changes in such parameters as the molar ratio (MR) of SDS/RuCl_3_, NaBH_4_/RuCl_3,_ or the type of stabilizer used on the Ru nanoparticles size and their size distribution was established.

- The effect of stabilizer concentration

**Figure 19:**
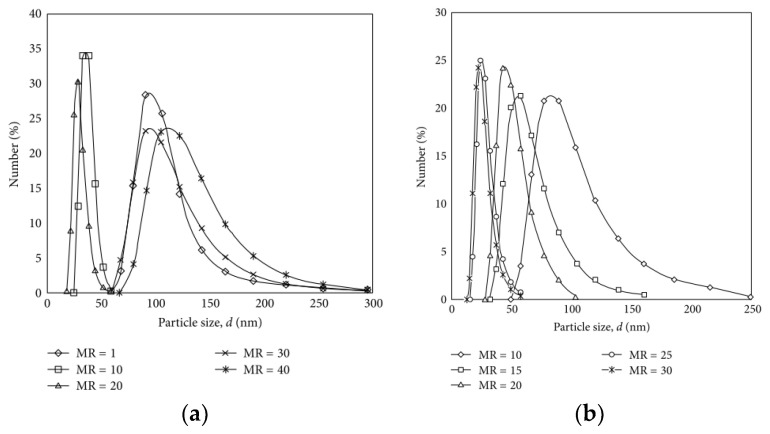
The effects of (**a**) SDS/RuCl_3_ MR and (**b**) NaBH_4_/RuCl_3_ MR on the particle size using a particle size analyzer. Reproduced with permission from [105].

The SDS/RuCl_3_ molar ratio was changed from 1 to 40, keeping the RuCl_3_ concentration at 0.2 mM and the NaBH_4_/RuCl_3_ molar ratio at 30. As shown in Figure 19a, the particle size decreased as the MR of SDS/RuCl_3_ increased to 20. The diameter of Ru nanoparticles was found to be 90 nm at MR = 1 and 20 nm at MR = 20. At MR > 20, the particle size increased as the SDS/RuCl_3_ ratio increased. The large size of the ruthenium nanoparticles formed at MR = 1 was interpreted as due to a higher degree of agglomeration, which was the result of an insufficient amount of stabilizing agent in the system. On the other hand, an increasingly higher surfactant concentration increased the viscosity of the system, which led to a decrease in the migration rate of the surfactant and/or a decrease in the diffusion rate of the micelles and a decrease in electrostatic repulsion, which had the effect of promoting the agglomeration process of the particles and so larger nanoparticles were ultimately formed.

- The effect of reductant concentration

The researchers decided to study the effect of NaBH_4_ concentration (MR NaBH_4_/RuCl_3_ = 10–30) on the size of Ru nanoparticles, holding other parameters constant (RuCl_3_ = 0.2 mM, MR SDS/RuCl_3_ = 20). The scientists observed that at a lower NaBH_4_/RuCl_3_ molar ratio (MR = 10), the size of the nanoparticles was larger due to insufficient reduction in RuCl_3_ (Figure 19b). However, as the molar ratio increased from 15 to 30, narrow peaks were obtained, suggesting that the Ru nanoparticles produced were smaller in size. Based on the results, the researchers concluded that a lower concentration of NaBH_4_ produces boron hydroxide through hydrolysis of NaBH_4_. The boron hydroxide was then absorbed into the Ru nanoparticles, reducing the electron density of the surface and causing the Ru nanoparticles to aggregate, resulting in a larger nanoparticle size. On the other hand, a higher concentration of NaBH_4_ increased the concentration of boron hydroxide, which formed a thick BH^4−^ layer, preventing boron hydroxide from being absorbed into the surface of Ru nanoparticles, resulting in well-dispersed yet smaller nanoparticles.

- The effect of stabilizer type

**Figure 20:**
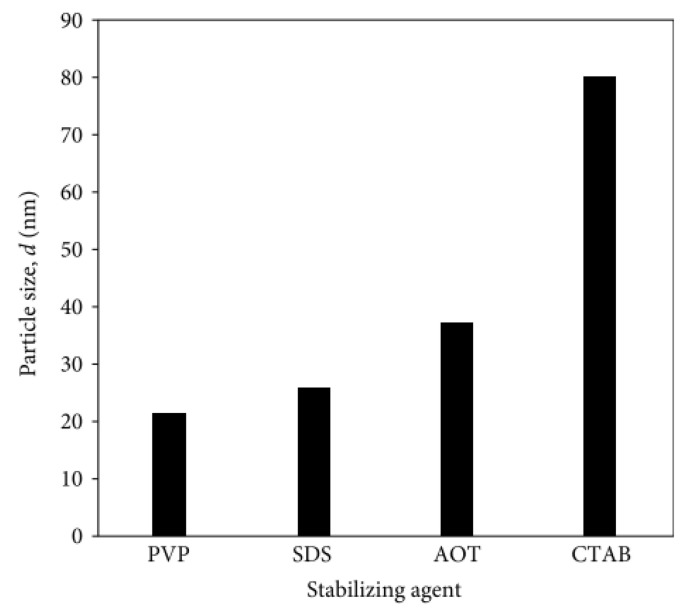
Study the effect of different types of stabilizing agents on particle size using a particle size analyzer. Reproduced with permission from [105].

In order to establish the effect of different types of stabilizing agents on nanoparticle size, a series of syntheses were performed in which PVP, SDS, CTAB, or AOT were used as stabilizers, and the final materials were subjected to particle size testing (RuCl_3_ concentration = 0.2 mM, MR surfactant/RuCl_3_ = 20, MR NaBH_4_/RuCl_3_ = 30). The smallest particle size was obtained when using PVP (~20 nm) and SDS (~25 nm), which was much smaller than that measured when AOT and CTAB were used as stabilizers (Figure 20). The explanation was that PVP, due to its structure, can act as both a stabilizer and a reducing agent, which resulted in the formation of particles of small size. In contrast, in the presence of CTAB, which is a cationic surfactant, the Ru nanoparticles were attracted to the positive charge of the surfactant, where they agglomerated near the outer surface of the micelles, resulting in the formation of larger nanoparticles.

## 4. Conclusions and Future Perspectives of Nanoparticles

In this review article, we have tried to introduce the reader to the basic issues related to the field of nanotechnology and transition metal nanoparticles. This technology is a growing field in the current global market and has a wide range of applications in every industrial sector due to its unique properties. The global market for this advanced technology is growing at a very fast pace, and thus, the use of nanomaterials and nanoproducts is expected to increase worldwide in the coming years. However, adverse effects of some nanomaterials on human health and the environment have been documented. Therefore, there is an urgent need to develop new approaches and standardized procedures to study the potentially hazardous effects of nanoparticles on human health and the environment. Nanotechnology has so far already played a key role in the development of various fields. In particular, nanoparticles are widely used in a number of branches of industry, from biomedicine to engineering, due to their unique size-dependent physical and chemical properties (e.g., high surface-to-volume ratio).

The use of nanotechnology in medicine is having a huge impact on human health in terms of diagnosis, prevention, and treatment of diseases. The development of metallic nanoparticles is very rapid and multidirectional, giving these materials the potential as new tools for future therapeutic drug delivery methods, especially in the treatment of cancer, inflammation, diabetes, and antiviral therapy [32]. Key issues that are associated with the clinical development of nanoparticles are biological difficulties, large-scale manufacturing, biocompatibility, and cost-effectiveness compared to current therapies [154]. Therefore, extensive research in the field of nanomedicine, especially in drug delivery systems, is urgently needed. The rapid development of nanotechnology provides practical tools for using this fascinating technology, especially in the aspect of biofuels. As already proven, the use of different types of nanoparticles—especially metallic ones, for the production of various types of biofuels (biodiesel, biohydrogen, biogas, and bioethanol) can improve productivity and production efficiency [155]. However, high production, separation, and reuse costs limit the practical application of biocatalysts for biofuel production. The use of a nanobiocatalyst can overcome the disadvantages, mainly stability and reusability, thus reflecting the importance of biomass-based biorefinery to make it cost-effective and sustainable [156].

With the development of nanotechnology, a tendency toward the miniaturization of various goods has strengthened. For this reason, technology has changed from semi to milli and from micro to nano. It will not be a surprise if picotechnology soon appears on the market, but this will not happen suddenly. At the current rate of nanotechnology development, almost all branches of engineering, from electronics and medicine to robotics, are expected to rely on it for their efficiency, durability, reliability, and reproducibility. Therefore, may dare to claim that nanotechnology is the engineering of the future. With all this said, it is important to remember that nanotechnology, which can provide us with a wide range of capabilities that must be used thoughtfully and responsibly, is a tool with the power to change the world into a better place to live in, only if it is used for good purposes.

## Figures and Tables

**Table 1:** Parameters affecting the morphology of metallic nanoparticles, based on [65].

Synthesis Parameters	Reaction Conditions
type and concentration of metal salts	reaction environment
type and concentration of stabilizer	temperature
type and concentration of reducer	pH
molar ratio of stabilizer to metal salt	stirring
molar ratio of reducer to metal salt	synthesis time

**Table 5:** Characteristic parameters of nanoparticles and the corresponding characterization techniques [11,146].

Entity Characterized	Suitable Characterization Techniques
Size (structural properties)	TEM, HRTEM, SEM, AFM, XRD, DLS, SLS, NTA, SAXS, EXAFS, FMR, DCS, ICP-MS, UV-Vis, MALDI, NMR, TRPS, EPLS
Size distribution	DCS, DLS, SAXS, NTA, ICP-MS, FMR, DTA, TRPS, SEM
Surface area, specific surface area	BET, liquid NMR
Surface charge	Zeta potential, EPM
Shape	TEM, HRTEM, AFM, EPLS, FMR, 3D-tomography
Elemental-chemical composition	XRD, XPS, ICP-MS, ICP-OES, SEM-EDX, NMR, MFM, LEIS
Crystal structure	XRD, EXAFS, HRTEM, electron diffraction
Concentration	ICP-MS, UV-Vis, RMM-MEMS, DCS, TRPS
Agglomeration state	Zeta potential, DLS, DCS, UV-Vis, SEM, Cryo-TEM, TEM
Chemical state-oxidation state	XAS, EELS, XPS, Mössbauer
Density	DCS, RMM-MEMS
3D visualization	3D-tomography, AFM, SEM
Optical properties	UV-Vis-NIR, PL, EELS-STEM
Magnetic properties	SQUID, VSM, Mössbauer, MFM, FMR, XMCD, magnetic susceptibility

AFM—atomic force microscopy, BET—Braunauer–Emmet–Teller, Cryo-TEM—cryogenic transmission electron microscopy, DLS—dynamic light scattering, DSC—differential scanning calorimetry, DTA—differential thermal analysis, EELS—electron energy-loss spectroscopy, EELS-STEM—electron energy loss spectroscopy with scanning transmission electron microscope, EPLS—electrophoretic light scattering, EPM—electrophoretic mobility, EXAFS—extended X-ray absorption fine structure, FMR—ferromagnetic resonance, HRTEM—high resolution transmission electron microscopy, ICP-MS—inductively coupled plasma mass spectrometry, ICP-OES—inductively coupled plasma optical emission spectroscopy, LEIS—low-energy ion scattering, MALDI—matrix-assisted laser desorption/ionization, MFM—magnetic force microscopy, NMR—nuclear magnetic resonance, NTA—nanoparticle tracking analysis, PL—photoluminescence, RMM-MEMS—resonant mass measurement with micro electro-mechanical systems, SAXS—small-angle X-ray scattering, SEM -scanning electron microscopy, SEM-EDX—scanning electron microscopy with energy dispersive X-ray spectroscopy, SLS—static light scattering, SQUID—superconducting quantum interference device, TEM—transmission electron microscopy, TRPS—tunable resistive pulse sensing, UV-Vis—ultraviolet-visible spectroscopy, VSM—value stream mapping, XAS—X-ray absorption spectroscopy, XMCD—X-ray magnetic circular dichroism, XPS—X-ray photoelectron spectroscopy, XRD—X-ray diffraction.

**Table 6:** The average zeta potential of platinum particles was obtained at various temperatures and pH values. Reproduced with permission from [83].

Zeta Potential Analysis (mV)		T = 25 °C	T = 40 °C	T = 50 °C
pH = 4	Pt + water	−14.5	−43.8	−29.4
pH = 7	Pt + water	−20.7	−50.8	−55.7
pH = 10	Pt + water	−78.1	−45.4	−52.4

## Data Availability

Not applicable.
